# Transformative Global Health Pedagogy: A Dinner Curriculum for Medical Students and Residents

**DOI:** 10.15766/mep_2374-8265.11044

**Published:** 2020-12-03

**Authors:** Peter J. Frank, Katherine E. Schreck, Alexis Steinmetz, Erik S. Carlson, Conrad Stasieluk, Brian Borah, Hannah Reiser, Lucia Garcia, Ruth Kafensztok, Brian Medernach, Daniel Palazuelos

**Affiliations:** 1 Resident, Cone Health Family Medicine Residency Program, Cone Health; 2 Resident, Department of Family Medicine, University of Minnesota Medical School/North Memorial Hospital; 3 Resident, Department of Urology, University of Rochester Medical Center; 4 Medical Student, Loyola University Chicago Stritch School of Medicine; 5 Resident, Department of Psychiatry, Loyola University Chicago Stritch School of Medicine; 6 Chief Resident, Department of Internal Medicine, University of Illinois at Chicago; 7 Resident, Department of Obstetrics and Gynecology, University of Chicago Medical Center; 8 Assistant Director, Center for Community and Global Health, Loyola University Chicago Stritch School of Medicine; 9 Assistant Professor of Public Health Sciences, Loyola University Chicago Parkinson School of Health Sciences and Public Health; 10 Infectious Disease Fellow, Department of Internal Medicine, Loyola University Medical Center; 11 Assistant Director, Hiatt Global Health Equity Residency, Brigham and Women's Hospital; Director of Community Health Systems, Partners in Health; Assistant Professor, Harvard Medical School

**Keywords:** Global Health, Health Equity, Global Health Pedagogy, Global Health Education, Dinner Curriculum, Resident Education, Public Health, Speaker Series

## Abstract

**Introduction:**

The Hiatt Residency in Global Health Equity program at Brigham and Women's Hospital partnered with Loyola University Medical Center and the Stritch School of Medicine to build and share an innovative global health dinner curriculum (GHDC) based on the methodologies of transformative learning theory. This educational approach encourages trainees to critically analyze their frame of reference and has the potential to create practitioners equipped to advance health equity.

**Methods:**

The GHDC explored broad global health (GH) topics through facilitated discussions with faculty and an experienced guest discussant over dinner. Medical students and internal medicine residents attended sessions based on their availability and interest. Participants completed surveys before and after every dinner. Comprehensive post-curriculum surveys were collected after participants had been involved for at least 1 year.

**Results:**

In 2017–2018, 98% of the 37 participants preferred the dinner-style learning session to a didactic-style lecture (97% of the 37 participants in 2018–2019). Eighty-five percent (2017–2018) agreed or strongly agreed that dinners provided them with new knowledge on a GH topic (92% in 2018–2019). Seventy-two percent (2017–2018) agreed that the dinner introduced them to a new potential mentor in GH (66% in 2018–2019).

**Discussion:**

The GHDC has been particularly successful in introducing participants to unfamiliar areas of medicine and new mentors. A second strength is its accessibility to medical students and residents. Its dependence on local resources allows versatility and customization; however, this trait also makes it difficult to prepackage the curriculum for interested institutions.

## Educational Objectives

By the end of this activity, learners will be able to:

1. Articulate their opinions in a discussion-based education format.
2. Show both short-term and long-term knowledge gains on individual session objectives.
3. Articulate a wider spectrum of career possibilities in global health.
4. Express renewed dedication to incorporating global health work into their careers.

## Introduction

The demand for global health (GH) education by health care trainees has increased exponentially in the last few decades, driving a surge in the development of GH programs in medical schools and residencies around the world.^1–6^ Although much has been published on curricular topics in attempts to standardize GH course content, efforts to define the ideal pedagogical approach have lagged behind.^7–9^ Detailed descriptions of educational frameworks and the rationale for using them, as well as research demonstrating their efficacy, are lacking from the GH educational literature.^7^

A decade ago, the Lancet Commission published a seminal article on health professional education that called for the instructional reform necessary for the type of transformative learning essential for advancing health equity in and between countries.^10^ Transformative learning is an educational theory that describes how trainees can move beyond simple knowledge acquisition and formation in order to deconstruct the hidden curriculum and reevaluate their worldviews.^11–13^ Students are no longer passive observers, as strict delineations of teacher and learner are muted through bilateral discourse that alters frames of reference.^11–13^ Learners are empowered to examine problems critically and envision alternate realities, making this an ideal educational paradigm for GH programs.^14–16^ Historically, many GH programs have been built into the traditional structure of medical training and rely heavily on didactic instruction by expert authorities and independent coursework. Educational competencies are emphasized over the less tangible outcomes of transformative learning.^7,15^ Therefore, there is a potential disconnect in GH education between typical formats and best practices for creating practitioners capable of leading foundational change, suggesting we critically reconsider how we train our GH workforce.^10,16–19^

The dearth of publications detailing transformative GH curricular approaches in *MedEdPORTAL* and elsewhere led to the development of an innovative global health dinner curriculum (GHDC) by the Hiatt Residency in Global Health Equity (GHE) program at Brigham and Women's Hospital (BWH). With the support of faculty from the GHE program, Loyola University Medical Center and the Stritch School of Medicine have integrated this curriculum into their GH programming. This publication outlines the curriculum and its specific educational approach and serves as an implementation guide for other academic institutions interested in this type of GH education.

The GHDC includes recognizable GH themes within a transformative educational framework. Sessions consist of facilitated discussions that are intentionally held outside of the medical institution at the homes of faculty or participants. Attendance is limited to small groups to ensure opportunities for effective and open dialogue. This nonconventional classroom provides an intimate space where experts are considered peers engaging in an exchange of experiences and ideas over a shared meal. The GHDC serves as an intellectually rigorous curriculum that minimizes the need for didactic lectures and the existence of the teacher-learner hierarchy. This facilitates opportunities for mentorship, community building, and open discourse, all of which are fundamental to transformative learning.

This publication also details the preliminary data regarding the acceptance of this learning format by trainees, as well as some of the specific successes and challenges faced by both BWH and Loyola, because they utilized the curriculum for slightly different audiences. Future data are needed to better understand the sustainability and long-term impacts of the GHDC as well as to compare it to other pedagogical approaches in GH.

## Methods

The GHDC was first piloted by the Hiatt GHE residency program at BWH. There, the core curriculum was established with the intention of exposing residents to a transformative form of GH education in a more intimate setting. With the support of faculty from the Hiatt GHE residency program, this experimental curriculum was later implemented at Loyola University Medical Center and the Stritch School of Medicine with the goals of expanding its impact to include undergraduate medical trainees and determining its adaptability to a given institution's resources and needs.

The GHDC operated outside of the formal undergraduate and graduate medical curricula and contained topics covering broad GH themes (outlined in Appendix A). Topics served as starting points for facilitated discussions with faculty and an experienced guest discussant over dinner in a more relaxed setting outside the hospital. We invited medical students and residents to attend sessions based on their availability and interest and set the cap at 12 trainees per dinner. To prepare the participants, we provided them with several pieces of easily digestible material to review. The dinner itself included an open dialogue on the given topic and a period of time for smaller conversations and networking.

In order to collect data on each individual session, we asked participants to fill out short surveys before and after every dinner. We collected longitudinal data from comprehensive post-curricular surveys given to students after they had been involved for at least 1 year. The use of these surveys changed over the course of the project, but the outline above is how they are currently used and what we have found to be most helpful. We analyzed data quantitatively and qualitatively.

### Guiding Principles for the Dinner Curriculum

#### Logistics

A team of three medical students in partnership with the assistant director of Loyola's Center for Community and Global Health worked to coordinate the logistics behind each dinner session. The timing of each session depended on the availability of the invited speaker, with the goal of holding one session every other month at a minimum. The program can be implemented at whatever pace is ideal for a given institution, and dinner sessions can be organized according to the specific interests of the trainees.

#### Choosing a topic

A comprehensive list of topics covered at Loyola and/or BWH over that past 2 years can be found in Appendix A. The topics were wide ranging and included areas such as GH research, physician advocates, correctional medicine, the opioid epidemic, traumatized communities, and gun violence. We selected these topics based on the expertise of the faculty at our institutions and on the particular social barriers to health care in our local communities. Our goal was to provide practical knowledge and opportunities with which learners could immediately engage. For this reason, we leaned towards topics covering universal themes that could be applied in both international and domestic contexts (e.g., preferring topics about inequity and health care system financing instead of malaria treatment and water sanitation initiatives).

#### Core versus supplemental curriculum

GH encompasses many fields and concentrations, a fact that presents opportunities as well as challenges. Acknowledging that individual institutions might or might not have access to presenters with unique expertise and experience, we delineated a core curriculum and a supplemental curriculum. The core curriculum covered broad themes in GH that we believed all institutions should consider incorporating into their own GHDC. Meanwhile, the supplemental curriculum consisted of deep dives into a few of GH's many subconcentrations. These supplemental dinner sessions could be incorporated into an institution's programming as desired. Given the specificity of the topics in the supplemental curriculum, these dinners could present a challenge when attempting to secure a knowledgeable speaker with relevant experience. However, the supplemental curriculum could be a fruitful addition to the core curriculum and serve to broaden trainees’ perception of GH.

#### Speaker invitation

We invited speakers to dinners based on their expertise and proximity to our institutions. Although there were many distinguished GH speakers outside the Chicago area, it was our desire to strengthen ties with local physicians and to provide practical opportunities and connections for learners. For example, at Loyola, one dinner included an emergency medicine physician who spoke about combating gun violence in the community. Another dinner involved an infectious disease physician employed at a local jail who spoke about his experience working in correctional medicine.

#### Pre-session resources

After determining a session topic and inviting the speaker, we curated resources for the dinner. We asked the speaker to contribute a relevant article or audio file, and then, coordinators gathered two or three additional resources. The pre-session resources typically included one journal article, one article from a nonacademic publication, and one audiovisual resource (e.g., TED talk, podcast). These materials were not intended to be comprehensive; rather, they were meant to be engaging and to provide the appropriate background with enough detail to lead into an informed conversation with a knowledgeable speaker.

#### Dinner location

The majority of the dinner discussions were held at the house of a student or faculty member with the understanding that transformative learning often takes place in environments outside of the conventional classroom. However, on the rare occasion that student or faculty homes were unavailable, we occasionally had to host the dinner session in a student lounge area at the medical school.

#### Participant invitations

Three to 4 weeks prior to each dinner, we sent out invitations to residents and medical students along with a short description of the session's topic and a biography of the guest discussant. One week prior to each dinner, we provided the curated resources on the topic to those residents and students who had expressed interest in the dinner. The specific resources we sent for each topic can be found in Appendix A.

#### Dinner structure

The majority of dinners lasted no longer than 2 hours. We typically began at 6:00 pm, with 30 minutes of meeting and greeting before dinner. This period provided an opportunity for residents and students to meet the guest discussant and mingle with each other. The mix-and-mingle period was structured to facilitate connecting people with a shared interest in global and community health. As we typically held dinners off the hospital campus in unfamiliar areas, the meet-and-greet period also provided extra time for participants to arrive. Shortly after the mingling period, we served food and began an hour devoted to discussion of the evening's topic. As an authority on the topic, the guest discussant had much knowledge and experience to share; the students and residents, though less experienced, were expected to contribute as well. A facilitator steered the conversation and elicited comments from both the guest discussant and participants. While it was tempting to let the guest discussant dominate the conversation, our goal was to facilitate learning through dialogue. Our participants had diverse GH experiences and often brought valuable comments to the conversation.

#### The power of good facilitation

Acting as a facilitator for these dinner discussions was not easy, but it was crucial. A few key points of advice for developing the skills necessary to facilitate effectively are articulated in the Art of Facilitation/Best Practices for Facilitators section of Appendix A.

#### Evaluation

Early in the implementation of the curriculum, pre- and post-dinner surveys were sent out via email before and after the dinners. In an effort to improve response rates, we transitioned from email to tablets available at the dinner. When participants arrived, we administered a short pre-dinner survey using an electronic tablet. This initial survey asked if the participant had been able to review all of the pre-dinner resources and inquired about the participant's level of comfort with the dinner topic. Before the participants left the dinner, they were given a short post-dinner survey using the same tablet. The post-dinner survey asked about knowledge gained, speakers and facilitators, session logistics, and overall impressions. These surveys provided information on each individual dinner and feedback on areas for improvement. Pre- and post-session surveys can be found in Appendices B and C, respectively. In order to track knowledge gained and impact over the course of many dinners, we asked participants to complete a comprehensive post-curriculum survey upon exiting the curriculum, which typically occurred 1 or 2 years after a participant's first dinner. This survey elicited information on GH knowledge, attitudes toward GH, vision for one's future career, and past service/travel experience.

## Results

From spring 2016 to spring 2019, we held 13 dinner sessions. Attendance was capped at 12 trainees per session, and attendance waxed or waned depending on the availability of trainees and their interest in the session's topic. From spring 2016 to spring 2018, 37 unique individuals (100% of all learners) completed our entrance survey and participated in the curriculum for at least one session (Table 1). Each learner filled out the entrance survey prior to attending the first meeting, and participants completed the exit survey after yearly curriculum completion. Although 100% of learners completed entrance surveys via email prior to the first dinner, we could only match 12 exit surveys to participants’ entrance surveys. We do not have data on the exact survey response rate from early in the implementation of the GHDC because attendance was initially not reliably recorded. The low response rate and challenges with keeping attendance records prompted a transition to a tablet-based administration of surveys, which resulted in nearly 100% of attendants registering their attendance and completing pre- and post-dinner surveys. In 2017–2018, 55 post-session feedback surveys were completed by trainees after attending a dinner. Halfway through 2018–2019, pre-session surveys were added to data collection to measure changes in self-reported objective knowledge. In 2018–2019, 41 pre-session surveys and 56 post-session surveys were completed. We successfully matched 37 pre- and post-session surveys in 2018–2019.

**Table 1.**
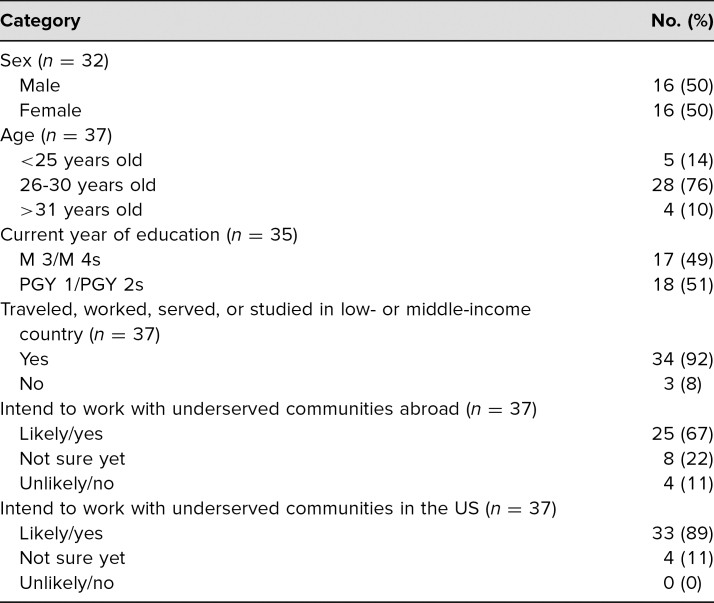
Demographic Information of Global Health Dinner Participants

Trainees highly preferred the casual, discussion-based format of dinners. In 2017–2018, 98% of participants preferred the dinner-style learning session to a didactic-style lecture (97% in 2018–2019), and the session timing worked well for 95% of participants (97% in 2018–2019). Participants enjoyed the discussion format, especially with the multiple perspectives present (Table 2). Ninety-one percent of participants thought that holding dinners in a faculty or student home was comfortable and contributed positively to the session. We found that a less formal, more relaxed environment allowed for more dynamic discussions and exchange of experience and information. The dinners held in the medical school often led to a less satisfying dialogue.

**Table 2.**
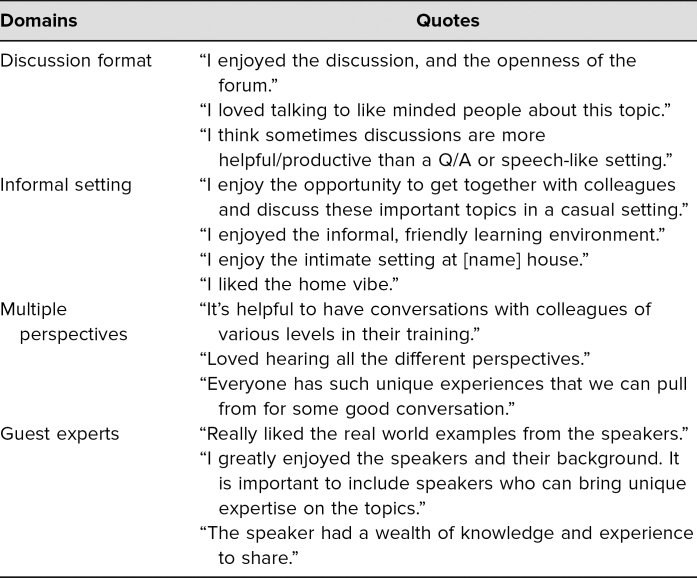
Themes Expressed by Global Health Dinner Participants

Pre-session assignments were generally useful when completed, but trainees could not always finish the assignments prior to the session. In 2017–2018, 89% of participants agreed or strongly agreed that pre-session assignments helped them prepare for the session (71% in 2018–2019), and 85% agreed or strongly agreed that pre-session assignments contributed to their understanding of the topic (72% in 2018–2019). As one participant said, “The pre-reading… gave me a foundation going into the discussion.” Interestingly, several participants cited pre-session assignments as one of the most useful aspects of dinners in comments, although other participants commented that they were not able to complete the assignments and therefore did not gain anything from them. Notably, in 2017–2018, only 35% of participants completed all the assignments prior to attending dinner (39% in 2018–2019), while 51% completed some to most (39% in 2018–2019). One participant mentioned, “I didn't watch the video or listen to the podcast because of time constraints.”

Participation in dinner sessions resulted in self-reported short-term knowledge gains, with no instances of self-reported short-term knowledge loss. In 2017–2018, 87% of participants agreed or strongly agreed that the dinners clarified concepts they could not learn on their own (82% in 2018–2019), and 85% agreed or strongly agreed that the dinners provided them with new knowledge on a GH topic (91% in 2018–2019). Surveys asked participants to self-rate on a 5-point scale their understanding of specific objectives unique to each session before and after attending (Table 3). Three to four objectives were provided per session. Examples included the following: “How would you rate your knowledge of the trauma-informed model of interviewing?” “Rank your understanding of climate change's indirect impacts on health.” “How aware are you of the health care barriers that the LGBT community faces?” Long-term knowledge gain data are currently under analysis.

**Table 3.**
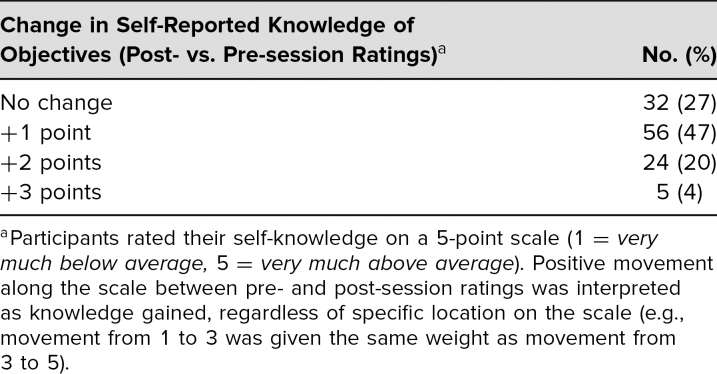
Matched 2018–2019 Survey Data From Global Health Dinner Participants

Trainees were able to identify new GH mentors through these sessions. In 2017–2018, 72% of participants agreed that the dinner introduced them to a new potential mentor in GH (66% in 2018–2019). Guest experts/speakers were overwhelmingly mentioned in participant comments as the most useful and informative aspect of dinners (Table 2).

Additionally, several trainees reported mentorship experiences with guest experts. In one instance, after attending a session on correctional medicine, a student became interested in the topic. This student contacted the guest expert and set up a clinical rotation with him at the Cook County Jail. As a result of this experience, the individual began considering a career in correctional medicine. In another instance, a guest expert offered mentorship for students entering family medicine who were also interested in GH. Several students engaged with her and received advice on how to search for residencies that would fit their particular interests. This physician also connected students with GH practitioners in their geographic areas of interest for residency.

## Discussion

The GHDC provided medical students and residents with opportunities to engage with GH topics in a format distinct from traditional didactic lectures, independent coursework, and online modules. The curriculum creates a space for sharing knowledge and experience where students and expert mentors are considered coinvestigators of major GH problems. Moving beyond merely informing trainees, this approach to GH education serves as an opportunity for transformational learning that potentially sparks their GH-related passions and sustains them into their future careers.

The GHDC was particularly successful in introducing participants to unfamiliar areas of medicine and new mentors. Medicine can take on myriad applications, many of which are not covered in the limited medical school clerkships. The GHDC presented new ways of meeting a community's diverse needs. These experiences came to students at a time when they were considering important career decisions regarding what field of medicine best suited them. Exposure to and engagement with potential career options were crucial for students to transition into an effective professional practice.^20^ GHDC students and residents who met or read materials from practitioners of fields not commonly presented as viable career options reported that the exposure was influential in their career decisions. Notably, the discussion on correctional medicine led one student to reach out to the speaker and eventually spend a fourth-year elective rotation at the Cook County Jail. This student considered future work in correctional medicine as a result of the GHDC. Additionally, other guest discussants have followed up with other students and residents to help inform and shape young medical careers.

A second strength of the GHDC was its accessibility to medical students and residents. Most residents were able to clear an hour and a half of time in the evenings when their clinical responsibilities were often lighter or more flexible. Medical students also had fewer commitments competing for their time in the evening. Since the curriculum fit into their schedules, busy trainees could engage as much as they were interested. The accessibility of the curated audio or video resources allowed busy learners to listen during a commute, as opposed to piling textbooks onto a lengthy reading list. One or two short, easily digestible, high-impact articles seemed equally attainable. Our learners were enthusiastic and engaged but had little time for additional daytime lectures or more nighttime reading. Our most positive feedback about the curriculum noted that its format could fit into the mold of an already demanding schedule.

The ability of the GHDC to take advantage of local resources was the trait that made it most difficult to generalize for other programs. The original BWH curriculum included multiple valuable topics that Loyola did not include because we did not have the same faculty available to facilitate those discussions. In the same way, we do not expect another institution to carry an identical curriculum because available resources will vary. This dependence on local resources is a strength that allows versatility and customization to a curriculum. Unfortunately, this trait also makes it difficult to prepackage an identical curriculum for interested institutions.

Our quantitative assessment of the effectiveness of the GHDC relied heavily on the entrance and exit surveys. The initial dearth of exit survey data presented limitations regarding the analysis of the GHDC, and we believe that this occurred because completion of the entrance survey was a prerequisite for admission to the dinners whereas the exit survey had no analogous motivation. By making the surveys easier to fill out (i.e., shorter and available via tablet at the dinners), we achieved 100% completion rates. Future implementations of this program should consider emulating this model of survey administration so as to maintain a consistent record of attendance and ensure high survey response rates.

The data show that students enjoy learning through the dinner discussion model, but we have limited objective measures to show what has been learned. Later in curriculum implementation, we modified pre- and post-dinner surveys to include learning objectives for each dinner. These surveys ascertained whether short-term knowledge was gained after attending a session. In late 2019, the program implemented an exit survey with learning objectives from sessions during 2018–2019 to identify any long-term knowledge gains. This analysis is forthcoming.

One challenge of the GHDC itself is fostering an intimate, honest discussion environment. A consistent, close-knit community allows a more relaxed environment with more open discussion. The original curriculum approached this by placing limitations on participation eligibility in order to create a more consistent collective group experience. The original curriculum also promoted team building by having the group take an international medical trip together at the start of the year. We at Loyola plan to acquire protected time off from resident responsibilities so as to encourage dinner attendance. The program requires only 2 hours of protected time every other month for residents enrolled in the GH track and can lead to a consistent group and a better learning experience.

The program at Loyola, inspired by more than 6 years of success at BWH, has seen positive outcomes in its 2-year history. As a young project, Loyola's program will benefit from adjustments to better achieve its goals and better quantify its accomplishments. Our aim in sharing our progress is to add to the body of literature on GH education and to encourage other institutions to consider incorporating a transformative dinner program into their GH curriculum with the ultimate goal of developing GH practitioners who are equipped to advance health equity.

## Appendices

GH Dinner Curriculum Manual.docxPre-session Survey.docxPost-session Survey.docx
All appendices are peer reviewed as integral parts of the Original Publication.
