# Supplementary material for: Transformative Global Health Pedagogy: A Dinner Curriculum for Medical Students and Residents
Source: MedEdPORTAL. 2020 Dec 3;16:11044. doi: 10.15766/mep_2374-8265.11044 (PMC7727609; doi:10.15766/mep_2374-8265.11044)
Supplement: Supplementary file 1 — GH Dinner Curriculum Manual.docxPre-session Survey.docxPost-session Survey.docx [file mep_2374-8265.11044-s001.zip › A. GH Dinner Curriculum Manual.docx]

GLOBAL HEALTH DINNER CURRICULUM MANUAL

Goals of the Program

1. Increase trainees’ knowledge of global health themes
2. Introduce trainees to potential mentors in global health
3. Expand trainees’ spectrum of understanding of what they assume to be possible in global health
4. Improve trainees’ ability to integrate global health into their professional careers

Methods to Achieve the Above Goals

1. *Provide informal (but serious), discussion-based educational experiences.* These are not lectures! Everyone involved should come with questions and the willingness to be an active participant in the discussions.
2. *Open a space that is conducive to good discussion.* Ideally, this will be in a student/faculty home (backup option could be a restaurant, preferably with comfortable couches in a corner where there will not be too much noise, etc.). These should definitely not be held in a lecture hall or anywhere with desks arranged in a traditional classroom style.
3. *Make this a communal meal experience.* Shared food opens the space for discussion and encourages community building (as opposed to a boxed lunch that implies you can leave unnoticed whenever you want). Ensure vegetarian/vegan options are available. Alcoholic and nonalcoholic beverages can be available if possible and acceptable.
4. *Easy prep for the busy resident or medical student.* Send out only highly digestible preparation materials. We want trainees to come to the session prepared with a working foundation of the topic, but we do not wish to create an additional (and unrealistic) academic load to their already busy medical school or residency experiences. The goal is to find a couple of informative resources that are not incredibly time-consuming, and fun to engage with, such as multimedia sources, podcasts, short videos or movies, etc.

The Art of Facilitation / Best Practices for Facilitators

The art of facilitating a conversation over dinner requires grace and charm, knowledge and humility. Some people are naturals, but anyone can learn. As with anything new, skill will improve with practice. This facilitator guide is meant to provide you with useful pointers on how to initially approach this challenge and continue improving with each dinner session.

1. Always take time to introduce the discussant.
   1. Perhaps start with, "tell us about yourself," and then extend an invitation for discussion by asking directed questions and prompting dialogue.
2. Help people feel good about *what* they’re learning by helping them feel good *while* they are learning.
   1. The facilitator should create an atmosphere where everyone feels welcome, comfortable, and joyful.
   2. The foundation for this includes learning participants' names as well as their motivations for attending the dinner.
3. Be comfortable with making people uncomfortable.
   1. Do not be afraid to ask questions that will move trainees out of their comfort zone and into a space of shared, constructive, and possibly challenging dialogue. Do this with a bright and generous affect, such as after you ask a difficult question, smile, raise your eyebrows, and tilt your head as if to say “OK, I know this is tough, but let’s get into it together.”
4. Guide the conversation without making participants realize that they are being guided.
   1. Allow the discussion to proceed naturally, and then chime in when there is a prolonged pause or if participants begin to veer off-topic.
   2. Some participants will talk more than others, and very active talkers can sometimes ruin the experience for others. Shutting them down publicly, however, can make the atmosphere tense and send the message this conversation is being micromanaged. The best conversations follow the form of being open, expansive, but always building.
   3. One useful strategy for getting quiet people to speak is to watch their eyes and when you see them react to a comment, take that opportunity to say: “[the person’s name], I see you may have an opinion about that? What are you thinking?”
   4. A strategy for getting big talkers to speak less is to approach them after the first dinner and say: “hey, thank you so much for adding this great energy to the conversation. I’m hoping that others will participate as much as you. What do you think I can do to get others to speak more?” Then, when they give suggestions, say: “Good ideas. I’m going to try that next session. To leave space for me to try these out, could you hold back some of your contributions? They are fantastic, but I want to leave some space open as you know some people are really shy.” The goal in these, or similar, statements is to recruit them into being a part of the solution instead of making them feel like they are a problem.
   5. Another strategy to try is to begin the first session by saying: “Our goal here is that everyone speaks for the same amount of time. We know this is nearly impossible because we are all very different people. You have full freedom to participate as you see fit. However, let’s do a thought exercise together: think of this space as a jungle with all sorts of wild animals that all live in harmony. Are you going to be a parrot that sings and sings and sings, but sometimes can’t be shut up? Will you be the jaguar that says nothing until you roar and attack? Will you be the monkey that never takes anything seriously, and is only here for the bananas? If we are going to live in harmony, just try to be conscious of how you are participating, and try to avoid being a beast!” Tell this story with humor, and allow for laughter (e.g. build the story until you get to the punchline about the monkeys only being there for the bananas as you point to the food and drinks). If successful, people will get the message and self-regulate. You may even find that they will regulate among themselves, saying something like: “Stop being such a parrot! Let the rest of us speak.”
5. There are no bad questions, only bad answers.
   1. Create an atmosphere where anyone can ask anything, and then facilitate so that different questions can spark different facets of a larger conversation, even if the questions asked can sometimes be simplistic and not necessarily going in the right direction.
6. If the participants did not prepare with the assigned materials, that does not mean that they are not prepared to participate.
   1. Don’t make participants feel bad that they didn’t do the work. NEVER put anyone on the spot to reproduce knowledge from the pre-reading.
   2. The materials you give them should never be a prerequisite for them participating in the conversation. These medical students/residents are paying with their time. If it becomes evident that when they do the work they will get more from their experience, then they will do the work. If you imply that if they don’t do the work they will not be welcome in the group, then they may avoid attending at all. If you are successful, they will see the preparation as something that they do to maximize their own experience.
   3. Consider taking 5 minutes before the dinner starts to explain the obviously main messages from the preparation. This is to get everyone on the same first step and to maybe inspire them to later go back and do the prep even if it is after the event.
7. Ask participants about their motivation for attending.
   1. Over the course of a session, take the time to learn participants’ names and ask participants about why they wanted to join the dinners. This will also help to coax the quieter members of the group because then you can pull them in based on what you know about them, such as: “[the person’s name], you have experience with this topic. I remember you once worked in Brazil. Does anything in your experience resonate with what we are talking about?”
8. Overall advice: relax, engage, and enjoy yourself. The facilitator’s mood sets the stage for the entire show.

Table of Contents

Core Curriculum

[Will Your Global Health Effort Make a Global Impact? 4](#_Toc38198732)

[The US Healthcare System 5](#_Toc38198733)

[Global Health Management and Health Care System Strengthening: Physician, Manage Thyself! 6](#_Toc38198734)

[Understanding and Overcoming the Complexities of Human Stratification 7](#_Toc38198735)

[So, You Say You Want a (Research Career) Revolution? 8](#_Toc38198736)

[The Academic as Advocate 9](#_Toc38198737)

[Traumatized Communities 10](#_Toc38198738)

[Healthcare in Conflict 11](#_Toc38198739)

[A Chip Off the Ol' Mentorship Block 12](#_Toc38198740)

[Determining the Determinants 13](#_Toc38198741)

[The Ring That Binds Them All (Global Health & The Media Machine) 14](#_Toc38198742)

[Research in the Time of Imperfect Governance 15](#_Toc38198743)

[The Untold Story of Global Health Career Development 16](#_Toc38198744)

[Global Governance - Who’s Driving and Where Are We Going? 17](#_Toc38198745)

[Universal Goals, Global Teams – Global health and Non-Communicable Diseases 18](#_Toc38198746)

[To Sustain, or Not to Sustain… 20](#_Toc38198747)

Supplemental Curriculum

[Health Care Delivery in Post-Genocide States 23](#_Toc38198748)

[The Medical Implications of the Second Amendment 24](#_Toc38198749)

[Trans Lives & Healthcare 25](#_Toc38198750)

[You Are On Indigenous Land 26](#_Toc38198751)

[The Opioid Crisis: How Did We Get Here and Where Do We Go? 27](#_Toc38198752)

[Human Trafficking 28](#_Toc38198753)

[Incarcerated But Still Human: The Practice of Correctional Healthcare 29](#_Toc38198754)

[The US Healthcare System: A Look at Possible Solutions 30](#_Toc38198755)

# Will Your Global Health Effort Make a Global Impact?

Session Description

Students hope that their careers are impactful, on par with the magnitude of challenges they see affecting the world. Big problems demand big solutions, but students often feel overwhelmed by either their options or lack of opportunities to do work that will have a more significant impact. This session helps students engage with staff who have worked in considering how key efforts can have significant effects when done well and at the right time.

Profile of speaker: Ideally, a mid- to late-career "change agent" in healthcare (medicine, public health, etc.) with experience seeking to address inequities in healthcare. The speaker should be able to relate stories of their successes as well as failures and would ideally be able to engage trainees wherever they are in their educational formation.

Questions for Facilitation

*Opening narrative: You are training as a health professional, and many of you were attracted to this field because you wanted to help make the world a healthier place. The purpose of this session is to encourage you to "think big" when faced with extremely complicated problems. In these conversations, we'll explore how to consider your role in global health, how to pick an effort for your career, and how to approach constructing a career that is both personally sustainable and substantial.*

- *To the discussant*: How did you choose to do what you have done in your career? In other words, out of all the options facing a student, how did you choose what you did?
- *To the discussant*: Having a significant impact often means changing a lot of things for the better, but sometimes there are unintended consequences that change things for the worse. Did that happen in your career? How did you navigate that?
- *To the group*: In the podcast "Going Big," the Harlem Children's Zone tried to improve the situation for kids living in poverty in Harlem by jumping over many traditional mechanisms for addressing poverty. Instead of focusing on young people in Harlem after they have been traumatized by poverty, the Harlem Children's Zone worked to prevent the damages of poverty happening in the first place. This was not a revolution; it did not distribute wealth nor directly address racism. Instead, it better prepared the children to navigate a world full of challenges. Is work like that enough?
- *To the group*: If you want to work in academic medicine, how do you avoid getting distracted away from what you think is impactful work by the demands of career development?
- *To the group*: What is the most impactful thing that a young physician "change agent" can do?

Student/Resident Preparation

1. Podcast: [This American Life Episode #364: Going Big](https://www.thisamericanlife.org/364/going-big)^1^ (Act 1 only) (33 minutes)
2. Article: [Addressing the “Global Health Tax” and “Wild Cards”: Practical Challenges to Building Academic Careers in Global Health](https://www.ncbi.nlm.nih.gov/pmc/articles/PMC4885528/)^2^

Example Speakers

1. [Daniel Palazuelos, MD, MPH](https://connects.catalyst.harvard.edu/Profiles/display/Person/63042) is a global health implementer-educator who holds positions at Harvard Medical School, the Brigham and Women's Hospital, and Partners in Health
2. [Amy Luke, PhD](https://www.luc.edu/parkinson/faculty/directory/faculty_detail.cfm?criteria=1365) is a Professor of Public Health Sciences at Loyola University Chicago’s Parkinson School of Health Sciences and Public Health

# The US Healthcare System

Session Description

Healthcare reform in the US has been an ongoing discussion for decades. As practitioners in the healthcare system, understanding the various debates for reform and remaining politically engaged is a necessary component of providing comprehensive care to one's patients. Students should have some mastery of the main questions at hand to be engaged participants for the discussion.

Profile of speaker: Someone who has experience in either activism for healthcare reform, administration of reforms in health systems (e.g., a vice president of clinical affairs or a CEO or CMO of a health system), or other expertise in the administration of healthcare systems.

Questions for Facilitation

*Opening narrative: As healthcare providers, we live in the US healthcare system every day. How much of its functioning do we really understand? Are the frustrations we and our patient's face just a "fact of life" or are they evidence of a broken system straining at the seams? The Affordable Care Act is one of the most successful, and controversial, attempts to fix our system to date, but it certainly isn't the first or last. What have we learned? Where are we going next?*

- *To the group:*
  - *Take a poll in the room*: Raise your hand if you think that the US healthcare system is broken.
  - For the people that raised their hand, why do you think it’s broken?
    - *(People often point to problems of inequity, high cost, medical errors, worse healthcare outcomes despite high spending.)*
  - For those that do not think the healthcare system is broken, why?
    - (*People often point to innovation, and how inequities can spark innovation.)*
  - If you had to wait an extra week for a surgery treating a benign condition, but by waiting for a week everyone including homeless people would have access to that surgery, would you wait a week?
    - *(For a number of Canadians, this is an acceptable trade-off. There are often waitlists for benign procedures, and some express being willing to wait so that others may also receive treatment.)*
- *To the discussant*:
  - In your career, can you describe to us how you have seen the healthcare system change? And do you think that change is for the better or the worse?
  - The US spends more than any other country per capita on health care. Is that because patients want too much healthcare, doctors prescribe too much healthcare, or that the business of medicine pushes us to consume too much healthcare? Is it that the prices are too high? What role do insurance companies, pharmaceutical companies, and hospital corporations play? Is it possible, or useful, to put blame on any of the stakeholders in this problem?
  - Regarding changes in the US healthcare system, what makes you despair? And what brings you hope?
  - *(If the discussant has experience with health systems outside the US)*: Can you tell us about the health system in X country? What lessons do we have to learn from there?

Student/Resident Preparation

1. Podcast: [This American Life Episode #391: More is Less](https://www.thisamericanlife.org/391/more-is-less)^3^ (60 minutes)
2. Podcast: [This American Life Episode #392: Someone Else's Money](https://www.thisamericanlife.org/392/someone-elses-money)^4^ (60 minutes)
3. Optional book: [Medical Lifeboat: Will There Be Room for You in the Healthcare System? (Howard Hiatt, 1989)](https://www.amazon.com/Medical-Lifeboat-Health-Perennial-Library/dp/0060915609)^5^

Example Speakers

1. [Claudia Fegan, MD](https://cookcountyhealth.org/senior_leaders/claudia-fegan/) is the Chief Medical Officer of the Cook County Health and Hospital System
2. [Howard H. Hiatt, MD](https://ghsm.hms.harvard.edu/faculty-staff/howard-h-hiatt) is a past dean of the Harvard School of Public Health (1972-1984) and co-founder and associate chief of the Division of Social Medicine and Health Inequalities at the Brigham and Women's Hospital in Boston

# Global Health Management and Health Care System Strengthening: Physician, Manage Thyself!

Session Description

The best leaders don't control people; they control themselves, first. People then follow their example. Managing yourself requires an active process of self-reflection and cultivating humility. It also requires learning from mistakes. This session is focused on helping students first recognize that if they aspire to be good leaders this process will be inevitable. They should also realize that it's not going to produce the expected results if they don't actually learn how to approach challenges correctly.

Profile of speaker: Someone that has worked in healthcare leadership, such as the chair of a medical program, the head of an NGO, the leader of a research lab, or someone who has been an executive of a healthcare-related organization.

Questions for Facilitation

*Opening narrative: Leadership in global health can be on a large scale (e.g., managing multiple organizations that are tackling an infectious disease outbreak), to the medium-scale (e.g., managing a regional team of community health workers), to the small-scale (e.g., managing a healthcare team in a clinic). Regardless of scope, there is a demand for strong, dynamic, competent, and compassionate leaders in global health that can navigate political, cross-institutional, and intra-institutional crises. This session will introduce us to leaders from different sectors who can offer their wisdom about navigating the challenges they have faced in their careers, which will hopefully be applicable to your own future leadership roles.*

- *To the discussant*: In your career, what is your proudest achievement?
- *To the discussant*: What were the challenges you faced while aiming to achieve that thing?
- To the discussant: The podcast NUMMI is a fascinating exploration of how a change for the better doesn't always happen even if it is a “good” change. Often there are people who don't want change for complex personal, emotional, or philosophic reasons. How have you worked through the various people-centered challenges that inevitably arise during your work?
- *To the group*: How do you achieve "good" management?
- *To the discussant*: How did you learn from your errors?
- *To the discussant*: In "See No Evil," they describe how the lack of a system of accountability led people in the program to act dishonestly. The people weren't inherently dishonest; they just accepted some unethical behavior because there wasn't a system in place to keep "honest people honest." What lessons have you learned from being an administrator that you wish you knew about when you first started?
- *To the group*: Let's say someone wants to be a good manager/leader. Where can people learn to do that? How does somebody know that they are on the path to being a good leader?

Student/Resident Preparation

1. Podcast: [This American Life Episode Episode #403: NUMMI](https://www.thisamericanlife.org/403/nummi-2010)^6^ (60 minutes)
2. Podcast: [This American Life Episode Episode #431: See No Evil](https://www.thisamericanlife.org/431/see-no-evil)^7^ (Act 3 Only) (10 minutes)
3. Article: [On Partnership](https://muse.jhu.edu/article/490780/summary)^8^
4. Article: [Internship in Africa: Death and Life](https://annals.org/aim/article-abstract/742567/internship-africa-death-life?volume=149&issue=5&page=353)^9^

Example Speakers

1. [Duncan Maru, MD, PhD](https://possiblehealth.org/who-we-are/team/duncan-maru/) is a cofounder of Possible Health, an organization which has built an integrated care delivery model that seeks to improve healthcare for the underserved of Nepal. He currently serves as a Senior Adviser and Board Member.
2. [Ryan Schwarz, MD, MBA](https://possiblehealth.org/who-we-are/team/ryan-schwarz/) is a Senior Adviser for Possible Health. His work focus on strategic and operational advising on Possible’s integrated health care delivery model, and partnerships and policy at the global level.

# Understanding and Overcoming the Complexities of Human Stratification

Session Description

Social stratification leads to different groups being exposed to different harms, and benefits. Discrepancies in these exposures lead to unequal consequences. A classic example of this is how African Americans in the US suffered from policies of redlining, which made it more likely for their neighborhoods to be poorer, more polluted, and more dangerous. They were socially stratified, which lead to increased exposure to harmful environmental factors such as pollution, and a lack of exposure to positive environmental factors such as peace, calm, accessible quality healthcare, etc. Thus, African Americans suffer a disproportionate amount of disease among Americans. The same can be said about immigrants in Europe, the indigenous in Latin America, the ‘untouchables’ in India, etc. Indeed, human beings seem to be drawn to separate out other human beings from their ranks, but If our goal in global health is health equity for all then this trend is something that needs to be explored further and better understood. Since this has been seen in almost every civilization, does this mean that this is just encoded in our DNA and therefore inevitable? Or, does knowing that there are some societies that are more tolerant and equitable than others give us hope that this should be understood as a human construct that can be counteracted, and reconstructed, with the right policies and programs?

Profile of speaker: The ideal speaker would be a medical anthropologist. Other potential speakers could be an anthropologist, any activist who works to reduce racism in medicine, or someone who works in law and/or reconciliation.

Questions for Facilitation

*Opening narrative: One of the core insights that drives many of us to work in global health equity is the realization that all humans on this planet are connected. This interconnectedness is due in part to the fact that we live in systems that have the potential to affect us all: climate change, the globalized economy, emerging diseases that don't respect borders, etc. Despite these powerful connections, many groups and countries still find ways to stay separate and in conflict. What drives this trend toward tribalism, classism, and dehumanization of other groups? Wherever we work, we will find the consequences of this reality. While some of our work will be in trying to battle against this trend's most toxic consequences, everything we do will have to take into consideration the fact that humans too often tend towards separation. During this dinner, we will explore together our experiences with this phenomenon, and discuss the methods we've discovered about how to navigate its most complicated negotiations.*

- *To the discussant*: Tell us about your work.
- *To the group*: Are humans genetically encoded to be tribal? And thus, is it inevitable that we will always have upper/lower class, favorable/unfavorable races, powerful/non-powerful genders? If not, why not? If so, then what do we do?
- *To the discussant*: How have you seen racism/sexism/fear of the other at play in your work?
- *To the discussant*: In your work, or in the work of colleagues, have you seen any efforts that are particularly successful in reducing discrimination? What about efforts that have fallen short of their goal? What can we learn from those failures?
- *To the group*: How can we, as current or future health practitioners, work to reduce the barriers to good health brought on by socially generated human stratification?

Student/Resident Preparation

1. Podcast: [This American Life Episode #491: Tribes](https://www.thisamericanlife.org/491/tribes)^10^ (Prologue and Act 1 only) (29 minutes)
2. Article*:* [Social Justice Should be a Key Part in Educating Health Professionals](https://www.statnews.com/2017/04/07/social-justice-health-education/)^11^
3. Article: [Is This How Discrimination Ends?](https://www.theatlantic.com/science/archive/2017/05/unconscious-bias-training/525405/)^12^
4. Article: [The Original Colonists, New York Times Book Review of: “The Social Conquest of Earth” by Edward O. Wilson](https://www.nytimes.com/2012/05/13/books/review/the-social-conquest-of-earth-by-edward-o-wilson.html?pagewanted=all&_r=0)^13^
5. Speech Transcript: [World Bank Group President Jim Yong Kim’s Speech at World Health Assembly: Poverty, Health and the Human Future](https://www.worldbank.org/en/news/speech/2013/05/21/world-bank-group-president-jim-yong-kim-speech-at-world-health-assembly)^14^

Example Speakers

1. [Evan Lyon, MD](https://humanrights.uchicago.edu/people/dr-evan-lyon) is the Chief Integrative Health Officer for the Heartland Alliance Health, a Chicago-based NGO serving people experiencing homelessness, mental illness, addiction, and chronic illness
2. [Ranu Dillon, MD](http://researchfaculty.brighamandwomens.org/BRIProfile.aspx?id=6584) is a physician and researcher focused on developing community-based primary health systems in low and middle-income countries

# So, You Say You Want a (Research Career) Revolution?

Session Description

“How to build an academic research career” is common material for any academic program. But the lessons commonly taught are not always applicable for research careers in global health. The best way to learn how to navigate that divide is through the tutelage of someone who has been through it themselves. You have to do it to know it.

Profile of speaker: Someone who considers themselves a global health researcher (MD, Ph.D., etc.), and ideally someone who has been successfully funded to do that research. If possible, invite more than one speaker, each with a different research background (e.g., implementation science researchers, bench lab researchers, infectious disease researchers, etc.).

Questions for Facilitation

*Opening narrative: Global health researchers face substantial challenges: funding for their work is low, the contexts where they work are unpredictable, long distances and regular travel can tax personal relationships at home. All this might lead you to ask: “is a research career in global health even possible?” Luckily, there are examples of people who have successfully developed such careers, and they provide for us possible pathways forward.*

*Traditional global health research careers have involved the study of infectious diseases. However, new and exciting fields such as implementation science, which seeks to apply rigorous analysis to the questions of how to best deliver healthcare to the world’s most vulnerable, are upending the traditional understanding of what a global health research career can be. Historically, the funding for research has come from either government sources (e.g. the NIH) or private resources (e.g., pharmaceutical companies), however, with new global health philanthropy (e.g., the Gates Foundation), there are now more options for finding the necessary funding for your work. Nevertheless, this is not always a straightforward process. This dinner will help you consider the best way to start and proceed.*

- *To the discussant*: Tell us about your research.
- *To the discussant*: What is your philosophy or reasoning behind why you do the research that you do?
- *To the discussant*: Tell us about how you secured your first grant and how that begot more funding to develop your research enterprise (be it a lab, group, etc.)?
- *To the group*: Can the challenges facing the poor and vulnerable be solved through an academic exploration of potential solutions? Or, are they too large and too multivariate to be reduced to a research question? (I.e., Are politics and business better vehicles through which to tackle the ravages of poverty?)
- *To the group*: Is it ethical to spend large sums of funding on a study in a poor context knowing that the same money could change many lives in the area?
- *To the discussant*: Name a few lessons you wish someone had told you when you were a student/resident/fellow?
- *To the discussant*: If you could do it again, would you? If yes, why? If no, why not?

Student/Resident Preparation

1. Article: [NPR Goats and Soda: Parachute Research](https://www.npr.org/sections/goatsandsoda/2016/04/02/472686809/scientists-say-its-time-to-end-parachute-research)^15^
2. Article: [Clinical Research and Global Health: Mentoring the next generation of healthcare students](https://www.ncbi.nlm.nih.gov/pmc/articles/PMC2958226/)^16^
3. Article: [Implementation Science](https://science.sciencemag.org/content/318/5857/1728)^17^
4. Example Article of Global Health Research: [The Lancet: Population Effect of Vaccines](https://www.thelancet.com/journals/langlo/article/PIIS2214-109X(14)70224-4/fulltext)^18^
5. Video: [NIH Tips for Applicants](https://www.youtube.com/watch?v=9cNRMsCGfHo&feature=youtu.be)^19^ (5 minutes)

Example Speakers

1. [Majid Afshar, MD](https://www.loyolamedicine.org/doctor/majid-afshar) is is a pulmonary and critical care physician and assistant professor in public health sciences at Loyola. He is currently a member of the Alcohol Research Group and Burn and Shock Trauma Research institute at Loyola.
2. [Serena Koenig, MD](https://researchfaculty.brighamandwomens.org/BRIProfile.aspx?id=6185) is an Assistant Professor of Medicine at Harvard Medical School. She devotes the majority of her time to conducting implementation research to improve health outcomes for patients infected with HIV and tuberculosis in Haiti, developing models for use in other resource-poor settings.

# The Academic as Advocate

Session Description

How can physicians affect policy and practice through their academic work? This often takes a significant effort that usually goes unreimbursed, so it is important for students to understand how to do this both effectively and logistically.

Profile of speaker: Someone who has been involved in policy change at a district, state, national, or international level. This could be someone who is a bureaucrat in a governing body (worked at the UN or WHO) or someone who has done work as an activist (e.g., [Dr. Mona Hanna-Attisha](https://monahannaattisha.com/) of the Flint, Michigan lead water crisis). If such people are not available, even a doctor who has written an op-ed will suffice.

Questions for Facilitation

*Opening narrative: If you decide to work in academic medicine, you will find that a large part of your promotion up the ladder to professor is based on publications. For many in global health, however, publications are only one tool among many to achieve our ultimate goals of increased health access and survival for the poorest populations. The heavy emphasis on publishing, however, can sometimes create an unfortunate pattern in which global health experts enter poor communities looking to achieve a preconceived goal. Does this make research just another neo-colonial extractive industry, taking resources from the global south (be it data or raw ore) that primarily can be used as currency in the global north (be it a publication or a gold ring)? Today we are going to discuss how to use a variety of tools to affect social change, including publications, and how to avoid unintended consequences.*

- *To the discussant*: Tell us about an experience you had where your efforts led to policy or practice change?
- *To the discussant*: What does it take to change policy and practice? What is most effective?
- To the discussant: If someone wanted to be an influential academician, changing policy and practice, what advice would you give them?
- *To the discussant*: Is there some advice you wish had yourself when you were starting?
- *To the discussant*: Sometimes even good programs can have unintended consequences. Tell us about a mistake you made when trying to enact change.
- *To the group*: One commonly given piece of advice is that in your academic, peer-reviewed work, “keep your writing tone scholarly and disinterested.” In your advocacy work, “keep your tone scholarly and passionate,” but don't mix the two. Would you agree with that advice?
- *To the group*: Is research just another extractive industry, taking resources from the global south (be it data or raw ore) that primarily can be used as currency in the global north (be it a publication or a gold ring)? If so, what can we do to avoid this unfortunate situation? If not, why not?

Student/Resident Preparation

1. Audio: [Eliminating TB: The Lancet, Oct. 26, 2015](https://www.thelancet.com/doi/story/10.1016/audio.2015.10.23.2404)^20^ (19 minutes)
2. Article: [Perspective: Physician Advocacy: What Is It and How Do We Do It?](https://journals.lww.com/academicmedicine/fulltext/2010/01000/Perspective__Physician_Advocacy__What_Is_It_and.22.aspx)^21^
3. Article: [The essential role of physician as advocate: how and why we pass it on](https://www.ncbi.nlm.nih.gov/pmc/articles/PMC5661729/)^22^
4. Optional Book: [Blind Spot: How Neoliberalism Infiltrated Global Health (Salmaan Keshavjee, 2014)](https://books.google.com/books/about/Blind_Spot.html?id=4vQTBAAAQBAJ)^23^

Example Speakers

1. [Julie O’Keefe, MD, FAAP](https://www.loyolamedicine.org/doctor/julie-okeefe) is a general pediatrician and has been involved in community pediatrics since 1999, mostly via the American Academy of Pediatrics Community Access to Child Health program
2. [Salmaan A. Keshavjee, MD, PhD,](https://ghsm.hms.harvard.edu/faculty-staff/salmaan-keshavjee) ScM is a professor in the Department of Global Health and Social Medicine and Department of Medicine at Harvard Medical School, and director of Harvard Medical School’s Center for Global Health Delivery–Dubai.
3. [Gordana Krkic, CAE](https://www.iafp.com/contact-us) is the Chief Advocacy and Policy Officer of the Illinois Academy of Family Physicians

# Traumatized Communities

Session Description

Healthcare providers are increasingly recognizing that substantial proportions of our patients have experienced trauma at some point in their lives. Trauma can take many forms, but what all these experiences have in common is that some, or even all, of the damages that occurred, are often hidden to us. This means that it is up to us to be better at recognizing the signs of possible trauma, and then to not re-traumatize the person as we’re working to piece together what happened so as to better help them. Treating traumatized patients, therefore, requires that we adapt our practices to their reality. The art of doing this is two-fold: first, wanting to do it correctly, and then, applying what is known to work to actually do it well.

Profile of speaker: Anyone who has been identified as a skilled practitioner of trauma-informed care (e.g., a social worker, a family medicine physician, a psychiatrist, a nurse, etc.).

Questions for Facilitation

*Opening narrative: Some populations within the United States have a particularly high prevalence of trauma during childhood, or what has been termed adverse childhood experiences (ACEs). Other populations may have experienced less trauma personally but still experience trauma from events that happened to their communities in a very different place or long before they were even born (the Tuskegee study is a key example, as those researchers withheld treatment for syphilis from a group of African American men for decades in the name of science, but their motivations were actually thinly cloaked in base racism). During this dinner, we will discuss how best to approach and interact with patients with a history of trauma.*

- *To the discussant*: Can you describe to us the different forms of trauma that you've seen in your work?

Can you describe to us what it means to provide trauma-informed care?

- *To the discussant*: Providing trauma-informed care is, as we said, an art form. There are a variety of reasons why a physician may not see this as a core part of their practice. What advice would you give them as a base minimum of what they need to do when caring for a traumatized patient?
- *To the discussant*: What do you know about the magnitude of the problem? Can you give us some statistics around trauma? In other words, how many of our patients are going to be traumatized without us even knowing it?
- *To the discussant*: When do you suspect a patient has a history of trauma? How do you proceed in caring for them?
- *To the discussant*: What works in helping patients overcome trauma? In other words, what works best to promote healing?
- To the group: Some forms of trauma are personal, and some are inherited (i.e., through the historic trauma of a community), which goes even deeper. How do we begin to work through that?
- *To the group*: When considering historical traumas of a population, some believe that time washes away the trauma and can no longer be considered an "excuse." What is your own opinion about that, and what do we say when someone confronts us with that opinion?

Student/Resident Preparation

1. Video: [TEDx: Beyond the Cliff - Laura van Dernoot Lipsky](https://traumastewardship.com/watch/#ted)^24^ (20 min)
2. Article: [45 years ago, the nation learned about the Tuskegee Syphilis Study. Its repercussions are still felt today.](https://www.usatoday.com/story/news/2017/07/25/tuskegee-syphilis-study-its-repercussions-still-felt-today/506507001/)^25^
3. Article: [Aces Too High: Got Your ACE Score? What’s Your ACE Score? (and, at the end, What’s Your Resilience Score?)](https://acestoohigh.com/got-your-ace-score/)^26^
4. Article: [The Little Known History of the Forced Sterilization of Native American Women](https://daily.jstor.org/the-little-known-history-of-the-forced-sterilization-of-native-american-women/?fbclid=IwAR2CF8JfbMw75nT9ISylujbYEm553d-UibI7r8mLDwBrj8MA75g51Se8gM4)^27^
5. Article: Trauma-Informed Care And Why It Matters^28^

Example Speakers

1. Hope Williams, LCSW is a Licensed Clinical Social Worker and Project Coordinator managing the Methamphetamine and Suicide Prevention Initiative (MSPI) grant at [American Indian Health Service of Chicago](http://old.aihschgo.org/)

# Healthcare in Conflict

Session Description

As long as human beings engage in conflict, people will get hurt. This may be obvious, almost foolish, to say but what follows is that healthcare providers will therefore always be needed when conflicts occur. Nevertheless, this doesn't mean that healthcare providers always provide the right support, act in the most useful ways, or even always make decisions that are in the best interests of their patients. This session will give students the opportunity to speak with someone who has firsthand experience with the complexity of engaging in healthcare during times of conflict. This is in the hopes that they can share their perspective on what should be done to provide the highest quality care possible, what should be avoided to prevent the unintended consequences of even our best intentions, and what are our responsibilities to the affected populations once the acute conflict is over but the damages remain.

Profile of speaker: Anyone who worked in conflict zones, either during or after conflicts, or worked in politics or activism around a specific conflict.

Questions for Facilitation

*Opening narrative: The acute needs of disaster and conflict victims are well covered by media attention, and often receive relief from humanitarian organizations. But what happens once the smoke has cleared? Many relief groups pack up and move out after the acute needs are met. However, these populations continue to experience chronic and noncommunicable diseases without the aid of local medical care.*

*One of the most famous organizations that provide healthcare to persons living in conflict zones is* [*Médecins Sans Frontières (Doctors Without Borders)*](https://www.doctorswithoutborders.org/)*. They are the pre-eminent experts on healthcare in acute conflicts. Other organizations like* [*Partners In Health*](https://www.pih.org/) *aim to take care of what they call “chronic disasters”: the health damages that occur because of smoldering poverty, vulnerability, isolation, etc. How do we best attend to the medical needs of populations that have experienced disaster or conflict in the acute setting, and how does that differ from the needs of such a population once the acute crisis is over?*

- *To the discussant*: Tell us about your work.
- *To the discussant*: How did you get into this line of work?
- *To the discussant*: What are your biggest concerns about providing care in conflict situations?
- *To the discussant*: What is commonly done well, and what is commonly done poorly?
- *To the discussant*: Did you often worry about your safety, and how did you cope with those feelings?
- *To the discussant*: What does a career in conflict medicine look like? What are the career options, and are their opportunities for career growth?
- *To the discussant*: What advice do you wish someone gave you when you were first starting out?
- *To the group*: When the conflict is over, what is the responsibility of the care providers who engaged in supporting people in conflict? In other words, the damages of the conflict will be felt for many years later; at what point can the care provider feel OK about moving away from the area?
- *To the group*: Many conflicts across the globe are caused by processes that either directly or indirectly benefit us, as citizens of the “rich world” and the global north. Examples abound, but one salient example is how we enjoy our smartphones every day but rarely consider the suffering caused by the mining of the materials used to make that phone. How do we live with that realization, and how does it change the way you think about this type of work?

Student/Resident Preparation

1. Podcast: [The Hidden Cost of War](https://www.msf.org.uk/article/podcast-hidden-costs-war)^29^ (27 minutes)
2. Video: [The New Barbarianism - Chapter 1 and Chapter 3](https://www.csis.org/features/new-barbarianism)^30^ (11 minutes)
3. Video: [What can we learn from the worst humanitarian crisis of our time?](https://www.youtube.com/watch?v=ISVxZZX1BDY)^31^ (20 minutes)

Example Speakers

1. [Zaher Sahloul, MD](https://medglobal.org/creating-a-world-without-healthcare-disparity/#team) is a Critical Care specialist in Chicago. He is the president and cofounder at MedGlobal, an NGO founded in 2017 by physicians experienced in humanitarian medicine to address the health needs of the world’s most vulnerable.

# A Chip Off the Ol' Mentorship Block

Session Description

Being a good mentor requires a set of skills that are best applied to someone who knows how to be a good mentee. What are the skills of a good mentor? What are the skills of a good mentee? To explore this, it is good to hear from someone who has experience with this type of relationship firsthand.

Profile of speaker(s): Someone who does global health work and has taken on a mentee that has resulted in fruitful outcomes. This is most likely going to be a research relationship (clinically related or not), but it could also be a clinical relationship or administrative relationship. Inviting both the mentor and the mentee is best for learning about both sides of the conversation.

Questions for Facilitation

*Opening narrative: Getting the mentorship you need to help build your career is not easy. And yet, there are people who enter into fantastic mentor-mentee relationships that are fruitful for both. How do they do it? What are the barriers, and how did they get over them? This dinner will focus on how to make mentoring relationships work. What does it take to be a good mentor? How can you be a better mentee? We will be looking to learn practical advice that you can use in your everyday life as you get mentored, or mentor others.*

- *For a mentee*:
  - Tell us about your work.
  - When did you first start to collaborate?
  - What has been the nature of your mentor-mentee relationships (e.g., research, administrative, medical, etc.)?
  - What have you found works best when considering how often to meet or how long and over what context? In other words, tell us about the logistics of your mentor-mentee relationship.
- *For a mentor*:
  - Tell us about your work.
  - What does it take to be a good mentor? What does it take to be a good mentee?
  - What are the limits of a mentor-mentee relationship? In other words, are there certain personal topics that you think are better left untouched? For example, is it ever appropriate for a mentor to give relationship advice to a mentee (even if that advice is intended to help the mentee find a work-life balance)?
- *To the group:*
  - Can anyone tell us about a bad experience in a mentor-mentee relationship? (no names, please)
  - Can anyone tell us about a good experience in a mentor-mentee relationship? (feel free to give names)

Do you think this mentor would be willing to mentor more people, such as some of the people here tonight?

Student/Resident Preparation

1. Article: [The 5 Types of Mentors You Need in Your Life](https://ideas.ted.com/the-5-types-of-mentors-you-need-in-your-life/)^32^
2. Video: [TEDx: Why Leadership and Mentorship Does Not Need to Fail Us](https://www.youtube.com/watch?v=mibnG8XeHlU&feature=emb_title)^33^ (18 minutes)
3. Article: [Training Toward a Movement: Career Development Insights From the First 7 Years of a Global Health Equity Residency](https://www.jgme.org/doi/10.4300/JGME-D-18-00213.1)^34^

Example Speakers

1. [Carole Diane Mitnick, Sc.D](https://ghsm.hms.harvard.edu/faculty-staff/carole-diane-mitnick) is an Associated Professor of Global Health and Social Medicine at Harvard Medical School. Her research centers on the improvement of clinical management and programmatic policy for tuberculosis and multidrug-resistant tuberculosis (MDR-TB) globally.
2. [Gustavo E. Velásquez, MD, MPH](https://ghsm.hms.harvard.edu/faculty-staff/gustavo-e-velasquez) is an associate physician in the Division of Infectious Diseases at Brigham and Women’s Hospital.

# Determining the Determinants

Session Description

The social determinants are a very powerful predictor of who gets sick, who gets healthcare, and how they respond to that healthcare. Health systems and health providers are increasingly recognizing the wisdom behind concerted efforts to address social determinants. But, young trainees do not always have clarity of how they can address those effectively. This session allows the trainees to gain exposure to someone who has experience addressing the social determinants.

Profile of speaker: Anyone who has developed a program or worked in some capacity to address the social determinants of health. For example, someone who has created food, housing, or transport support program at a clinic. Someone who has worked with policymakers to develop policies that affect patients or patient outcomes. Someone who has engaged in a medical-legal partnership.

Questions for Facilitation

*Opening narrative: The illnesses we care for in the hospital and clinics where we work are the products of countless, often social processes coalescing into clinical entities. Our training in medical school and residency aims to make us experts in curing or palliating, but we know from our global health work that there is also much we can do "upstream" to influence who gets sick and how sick they get. The question for this conversation is: "ok, but how?" Even though we know that much of what makes our patients sick is determined by the structures they live in, we ourselves are structurally pushed towards focusing downstream. How do we actually become an "upstream" doctor"? What personal and professional opportunities, what formal or informal mechanisms, are available to us as we start paddling against the current?*

- *To the discussant*: Tell us about your work?
- *To the discussant*: Why did you feel that it was important to go upstream?
- *To the discussant*: A lot of the workflows of the US Health system are based on how we reimburse certain things over others (i.e. medical interventions over prevention and wellness). How did you find the funds necessary to do the work you wanted to do?
- *To the discussant*: If you could do this again how would you do it differently?
- *To the discussant*: What’s next for your work?
- To the group: Do you know of any projects or work that, to you, exemplify the work of addressing the social determinants of health at its best?
- *To the group*: In the NEJM Social Medicine article, the young doctor working at a clinic falls into "misrecognition" and then develops "critical consciousness." Misrecognition is failing to see that a patient's problem is heavily dependent on the situation in which they are living. What do you think are the main causes of "misrecognition" either in the US or abroad?
- *To the discussant*: What advice would you give our trainees that you wish you had gotten early in your career?

Student/Resident Preparation

1. Video: [What Makes Us Sick? Look Upstream](https://www.ted.com/talks/rishi_manchanda_what_makes_us_get_sick_look_upstream?utm_campaign=tedspread&utm_medium=referral&utm_source=tedcomshare)^35^ (18 minutes)
2. Article: [Structural competency: Theorizing a new medical engagement with stigma and inequality](https://www.ncbi.nlm.nih.gov/pmc/articles/PMC4269606/)^36^
3. Article: [NEJM: Misrecognition and Critical Consciousness — An 18-Month-Old Boy with Pneumonia and Chronic Malnutrition](https://www.nejm.org/doi/full/10.1056/NEJMp1902028)^37^

Example Speakers

1. [Sara Selig, MD, MPH](https://primarycare.hms.harvard.edu/faculty-staff/sara-selig) is an Associate Physician in the Division of Global Health Equity at Brigham and Women’s Hospital and an Instructor of Medicine at Harvard Medical School. Dr. Selig currently serves as the Associate Director for the Community Outreach and Patient Empowerment Program, the domestic affiliate of Partners in Health.

# The Ring That Binds Them All (Global Health & The Media Machine)

Session Description

Global health and global populations, including in the global north, are facing a wide host of problems. What receives attention depends on a complex combination of factors that do not necessarily follow a logical algorithm but yet have a logic of their own. This session will allow trainees time to reflect with someone who has experienced the shifts of publicity in a global health topic. The goal is to help trainees consider how to navigate being thrust into a publicity opportunity, what to do well, and what mistakes to avoid. In this scenario, the ring that binds them all is the multinational, publicity machine that exists because of social media. Without a better barometer of what is true, the truth has become what is said, is repeated, and “feels right” even if there is no evidence to back that claim. This is a mechanism that is powerful when understood, but when used incorrectly it can distract from more necessary work.

Profile of speaker: Anyone who has experience in working with the media and/or has achieved some media recognition for their work.

Questions for Facilitation

*Opening narrative: Global health often hits the public in waves of publicity, especially during times of crisis, such as when a new pandemic threatens the global north. As global health practitioners, we sometimes get caught up in that wave. During this dinner, we'll have the opportunity to enter into an intimate conversation exploring what it feels like to ride a meteoric rise in global health publicity, and how to use this momentum for the benefit of the movement for health equity.*

- *To the discussant*: Tell us the story of when and how you found yourself in the media.
- *To the discussant*: Do you feel that you “deserved” the attention you were getting?
- *To the discussant*: What did you do well? What mistakes did you make?
- *To the discussant*: If you could do it again, what would you do differently?
- To the discussant: Do you feel that you made the most of the opportunity? If so, how? What could you have done differently?
- *To the group*: A lot of times, opportunities that are presented to a group are actually the product of the work of many. How do you balance using the opportunity to maximize the work that you are trying to do as an individual vs. the opportunities that were made possible because of the work of so many others? In other words, how much should we focus on building our own brand vs. building the movement?
- *To the discussant*: Anyone who has achieved some fame will describe how there is their public persona and then who they really are. Can you reflect on the divide between those two poles? (If asked for clarification, you say that perhaps there is a spectrum between what the public sees and thinks and what is the full truth; this question is looking to explore the space between those two positions).

Student/Resident Preparation

1. An article or video that shows this discussant’s engagement with the media (below are examples that pertain to one discussant at Harvard Medical School).
2. Article: [Global Health and Journalism Look for Ways to Save the World in a New Media Landscape](https://pulitzercenter.org/blog/global-health-and-journalism-look-ways-save-world-new-media-landscape)^38^

Example Speakers

1. [Raj Panjabi, MD, MPH](https://lastmilehealth.org/team/founders/) is the CEO of Last Mile Health, and Assistant Professor of Medicine at Harvard Medical School and the Division of Global Health Equity at Brigham & Women’s Hospital
   1. Video: [TED: No One Should Die Because They Live Too Far From a Doctor](https://www.ted.com/talks/raj_panjabi_no_one_should_die_because_they_live_too_far_from_a_doctor)^39^ (20 minutes)
   2. Website: [Last Mile Health](https://lastmilehealth.org/chacademy/)^40^
   3. Website: [Community Health Impact Coalition](https://chwimpact.org/)^41^

# Research in the Time of Imperfect Governance

Session Description

It is not easy to run a research program in a fragile context. Such contexts may be in the throes of conflict, recently recovering from a conflict, or just simply impoverished and only slowly developing. Conducting studies there, however, is important to help expend the benefits of science to that area’s growth and recovery. Beyond being merely logistically difficult, other questions inevitably arise about what work is the most necessary actually fix the factors that contribute to that context’s fragility in the first place; this brings up the question of priorities and tactics for social change. Research has a role to play, but the global health researcher should understand how their work fits within a larger spectrum of activity that can be done to help lift the situation out of its current challenges. These questions are often seen as outside of the purview of science, but the scientist ignores them out of their own peril, especially if they aspire for their discoveries to fuel a broader and more lasting impact.

Profile of speaker: Someone who has conducted rigorous research in a fragile context. This could be the inner-city US, or a resource-poor setting during war, coup, famine, natural disaster, etc.

Questions for Facilitation

*Opening narrative: Once you have the funding, the mentor, and the space to conduct research, what do you do with unanticipated challenges? For example, how do you keep an implementation trial randomized in the time of a coup? Are food riots a threat to academic rigor? Where does promotion fit within the Ebola epidemic?*

- *To the discussant*: Tell us about your research.
- *To the discussant*: Tell us about how the context where you work is fragile? What are the factors that most contribute to its fragility?
- *To the discussant*: How have you practically overcome the challenges that arise because of this region's fragility?
- *To the discussant*: Doing research in less fragile contexts would arguably be easier. Why did you not choose the easier path for your research career?
- *To the discussant*: What do you think of this criticism: colonialism was an economic system that extracted raw materials from the global south for processing in the global north (with the goal of primarily increasing the wealth of those who controlled the processes of extraction). Most envision minerals, wood, and water when describing this process, but others would include data; does the process of extracting data from a poor context to process into publications in northern academic institutions now follow the same line of reasoning? Can you reflect on this tension? What do you do to minimize the potential negative side of a research agenda in a context that gets to many different needs?
- *To the discussant*: Some people say that the best way to perform research that extends across research, race, class, etc. is to pair that research with a capacity-building program. What are your thoughts on that? How is this done best? What are the best examples that you think others should emulate?
- *To the group*: what are other ways that you can envision to avoid the unintended consequences of research in poor contexts and with impoverished populations?

Student/Resident Preparation

1. Podcast: [This American Life Episode #410: The Social Contract](https://www.thisamericanlife.org/410/social-contract)^42^ (60 minutes)
2. Article: [The Tension in Business Education between Academic Rigor and Real-World Relevance: The Role of Executive Professors](https://www.jstor.org/stable/40214502?seq=1#page_scan_tab_contents)^43^
3. Article: [Building a Research Career in General Internal Medicine: A Perspective from Young Investigators](https://www.ncbi.nlm.nih.gov/pmc/articles/PMC1496912/)^44^
4. Article: [What Is an Academic General Internist? Career Options and Training Pathways](https://jamanetwork.com/journals/jama/fullarticle/195442)^45^

Example Speakers

1. [Louise Catherine Ivers, MB, BCh, MD, MPH, DTM&H](https://ghsm.hms.harvard.edu/faculty-staff/louise-catherine-ivers) is executive director of the Massachusetts General Hospital Center for Global Health, an associate professor of global health and social medicine and associate professor of medicine at Harvard Medical School, an associate physician in the Division of Infectious Diseases at MGH, and an associate physician in the Division of Global Health Equity at Brigham and Women's Hospital.

# The Untold Story of Global Health Career Development

Session Description

Global health work is challenging logistically and therefore requires special considerations on how to find the proverbial “work-life balance.” This is all the more true when hoping to build a family. This is not only because of the logistical challenges inherent in travel and living in resource-poor settings that offer fewer employment opportunities for spouses, and fewer educational opportunities for children; there also remains the question of how the global health practitioner feels about the risk for both themselves and their loved ones. Compounding this challenge is how friends and family feel about risk, and whether this leads them to express their opinions as either supportive advice or harsh judgments.

Profile of speaker: This session exposes trainees to someone who has attempted to balance global health work with their familial responsibilities, someone who has lived abroad for an extended period of time, potentially with partner and children in tow. Ideally, this person should be someone who can offer sage advice and help normalize the notion that it is indeed possible (while also being honest about the pitfalls to avoid, and the tough decisions that need to be made with limited information but incredibly high stakes).

Questions for Facilitation

*Opening narrative: In the podcast for this dinner, you heard the story of a family who went on a boat ride with their young children and ran into trouble needing an emergency rescue. What happened thereafter was a societal opinion trial, waged on social media platforms like Facebook, discussing whether or not these people were "good parents." Some did not think so, and this family still receives messages from strangers "hoping that they drown" for being such bad parents.*

*Global health career development is not only about who is funding your research, and what type of shift you are working in the US; it may also be about how you structure your family and personal life so that your actions can align with your ethics. What do you do when your peers, family, or even strangers feel obligated to opine about your choices? How do we maintain and grow meaningful relationships with those we love when our work is often defined by challenges like distance, travel, risk and even sacrifice? What is a reasonable risk to assume, for us, for our families? None of us went into global health to be bad people that make bad choices - quite the opposite. Nevertheless, there are better and worse ways of negotiating personal questions in global health career development, and the associated life choices. There are no easy answers - as a community of practitioners, let's learn from each other.*

- *To the discussant*: Tell us about your global health work.
- *To the discussant*: Tell us about your family.
- *To the discussant*: What considerations have you had to keep in mind when balancing your familial responsibilities with your global health aspirations?
- *To the discussant*: Tell us a story that exemplifies the *benefits* of including your family in your global health work.
- *To the discussant*: Tell us a story that exemplifies the *challenges* of including your family in your global health work.
- *To the group*: What do you do when people tell you that you are making the wrong choices?
- *To the discussant*: What advice would you give about keeping your family safe and happy when traveling abroad?

Student/Resident Resources

1. Podcast: [This American Life Episode #525: Call for Help](https://www.thisamericanlife.org/525/call-for-help)^46^ (Prologue, Act 1 and Act 2) (41 minutes)

Example Speakers

1. [Joia Stapleton Mukherjee, MD, MPH](https://ghsm.hms.harvard.edu/faculty-staff/joia-stapleton-mukherjee) is associate professor of medicine in the Division of Global Health Equity, Department of Medicine, Brigham and Women's Hospital, and associate professor of global health and social medicine at Harvard Medical School. In the Department of Global Health and Social Medicine, she directs the [Master of Medical Sciences in Global Health Delivery](https://ghsm.hms.harvard.edu/education/master-medical-sciences-global-health-delivery) program and the [Program in Global Medical Education and Social Change](https://ghsm.hms.harvard.edu/programs/medical-education).

# Global Governance – Who’s Driving and Where Are We Going?

Session Description

Engaging in global health can be overwhelming for trainees. Whatever sole issue initially attracted them, most will quickly learn that their favorite issue is only one piece of a much larger puzzle. This session is a more open exploration of what constitutes the "right work" in global health in order to be the most impactful. To help structure this conversation, we focus primarily on the structures of global health governance both to understand the processes at play for setting the global health agenda, and what is currently included in, or excluded from, that agenda. We’ll then turn our focus on questions surrounding accountability and one very challenging question: when most of our patients suffer from the abuses of unbridled capitalism, and we need donations from the profits of such businesses to run programs, where is the line between being an agent of change versus an accomplice?

Profile of speaker: Anyone who has thoughtfully engaged with global health governance or priority setting. This could be an activist who has tried to influence the global agenda, a reporter who has reported on deficits in the global agenda, a bureaucrat who helps multinational bodies (e.g., UN, WHO) implement its agenda, etc.

Questions for Facilitation

*Opening narrative: Global health is a field that is comprised of a wide array of overlapping disciplines and institutions. Among other reasons, the field began because of the wide array of overlapping risk factors that affect our patients' health. We, as individual global health professionals, need to navigate these complicated waters and make decisions about where to focus our efforts. In setting our personal and professional priorities, questions arise: what is the best work that will yield the best results? With whom should we work? What should be our ultimate goals? And, how do we, and our funders, stay accountable to our commitments and values?*

- *To the discussant*: Tell us about your work in global health?
- *To the discussant*: How did you choose this work over other work?
- *To the discussant*: Have you ever come across a situation where a proposed solution felt to you like it was actually a part of the problem? Examples might be working with a government that some criticize as being the cause of much suffering, receiving funding from a donor whose business practices are not entirely benign, or working in a vertical program to address only one problem when you know that the larger problem is actually a conglomerate of many different issues. What did you do?
- *To the discussant*: Most NGOs need donations to function. Ideally, they would only accept “clean money.” Does clean money actually exist?
- *To the group*: Drawing the line between what is perceived as harmful vs. beneficial is very subjective. Stories abound of how some funders are quite complex, but most will agree lean towards the positive (i.e., Bill Gates?). Other funders are clearly quite harmful for society and are using donations simply as a means to clean up their public image for past ills they have committed (i.e., the CSR wings of large corporations that abuse labor laws and bribe government officials to get favorable contracts, but then donate to a small water campaign and use the publicity to overwhelm any negative press). How do we, as future global health practitioners, develop the judgment of where this line should be drawn?
- *To the group*: Are the current systems of global governance sufficient for the global problems we face today? Is the WHO, PAHO, World Bank, etc. doing enough to tackle problems that cross national borders, such as climate change, pandemics, nuclear proliferation, terrorism fueled by extreme poverty, drug and human trafficking, etc.?
- *To the discussant*: Could you give examples of where global governance, as it exists today, has been sufficient? Can you give examples of where it has been woefully insufficient?

Student/Resident Preparation

1. Article: [Governance Challenges in Global Health](https://www.nejm.org/doi/full/10.1056/NEJMra1109339)^47^
2. Podcast: [Global Health Governance: Who is Accountable to Whom?](https://podcasts.apple.com/gb/podcast/global-health-governance-audio/id390466006)^48^ (1 hr and 44 minutes)
3. Article: [How McKinsey Infiltrated the World of Global Public Health](https://www.vox.com/science-and-health/2019/12/13/21004456/bill-gates-mckinsey-global-public-health-bcg)^49^
4. Video: [Anand Giridharadas - “Winners Take All” and the Paradox of Elite Philanthropy | The Daily Show](https://www.youtube.com/watch?v=H32z45o0WxA)^50^ (9 minutes)

Example Speakers

1. [Sam Loewenberg](http://www.samloewenberg.com/biography.html) is a journalist who covers the intersection of global health, business, government, and politics
2. [Mara Kardas-Nelson](https://marakardasnelson.com/) is a journalist who covers the intersection of health, environment, development, politics, and change

# Universal Goals, Global Teams – Global health and Non-Communicable Diseases

Session Description

Addressing non-communicable diseases (NCDs) and cancers have not traditionally been included in the toolbox of global health practitioners, but this is changing as the global health community increases its focus on non-infectious diseases. As the horizon expands, what are the opportunities which practitioners should look out for, and what are the pitfalls to avoid?

Profile of speaker: Someone who is working in global health, but not in HIV, malaria, etc. per se. The ideal discussant will be anyone who has worked with NCD disease programs in a global health context abroad as an activist, researcher, clinician, administrator, etc.

Questions for Facilitation

*Opening narrative: Global health traditionally focused on “diseases of the global south," which have for decades been infectious diseases. The roots of this are not always pretty; there was a distinct colonialist agenda to focus on such diseases so as to prevent contagion of the global north and to maximize the productivity of workers in the global south. One can see this persist today when pandemics, such as the Ebola crisis in West Africa of 2014-16 was ignored until it threatened to migrate beyond those borders. As global health has begun to expand in its scope and ambition, there is an opportunity to right these historic wrongs. Ironically, many NCDs are actually caused by risks that flow in the opposite direction, from north to south: air pollution from factories owned by global north corporations, big junk food and tobacco companies that push into new markets, global warming that is caused primarily by consumption in the rich world but felt most acutely by the rural poor, etc. Global health financing has not yet risen to address the growing burden of disease caused by non-infectious diseases such as diabetes, cancer, heart failure, or mental health conditions, but there is ever-increasing attention to these issues. If “universal health coverage” is successful in adding more seats to the table for these conditions, then we need to be prepared with how we’re going to deliver effective care, probably through a variety of teams all coordinating and/or working in unison.*

- *To the discussant*: Can you describe for us the burden of disease of NCDs globally as you have seen it from the level of your work?
- *To the discussant*: Preparation for this dinner included looking at some statistics. We are also interested in hearing what it looks like from the experience of impoverished people who are living these statistics. Can you tell us a story from your experiences in the field?
- *To the discussant*: Why is it currently difficult to adequately address NCDs in developing nations?
- *To the group*: What do you think needs to be done to improve this situation?
- *To the discussant*: Cost-effective analysis has historically devalued investing in the health systems that are best equipped to address “the long tail” of the NCDs (this means the large number of conditions that not be singularly a major cause of morbidity, but when taken as a whole amount to considerable morbidity and mortality). As the burden of disease increases and the cost of essential medications decreases, these analyses will change. But not every non-infectious disease will make the cut immediately, especially certain types of cancer. This suggests that a new way to frame the case for treating such patients will be necessary. In your experience, what works best in this advocacy?
- *To the discussant*: Can you describe some more of the logistical challenges for taking care of NCDs in impoverished nations, rural areas, etc.?
- *To the discussant*: For the people who care about NCDs, finding funding for this work, either as researchers, clinicians, or program developers, has notoriously been difficult. What do you think works best to develop a career in this field as this landscape changes and opportunities arise?
- *To the discussant*: Historically, funding for global health work has been focused on infectious diseases with the understanding that by funding the treatment and research of infectious diseases, which can cross borders, a country will be protecting the health and security of their own citizens. The neglected tropical diseases, such as Chagas disease, represent a case in point; since they do not cross borders, research working to address them and care for the people who have them is woefully inadequate. At the same time, NCDs are now getting a lot of attention. How does one capitalize on this increased momentum and avoid gimmicks/fads? An example of a gimmick or fad might be a specific programmatic design, a technology, or a pharmaceutical that promises to “revolutionize the field,” but it is quickly found to be obsolete, insufficient, or irrelevant.
- *To the discussant*: For the groups that work in high-prevalence well-funded diseases, such as HIV, they have traditionally not needed to collaborate with other groups to get their jobs done. The stories of how difficult it was to get the HIV programs to collaborate with the Tuberculosis programs are legendary. NCDs, however, face an entirely different situation. How have you seen collaborations in NCD work, or not work? What advice would you give these students as they work to find good teams, and effective collaborations?

Student/Resident Resources

1. Webpage (for browsing): [NCD Synergies - Health Equity for the Poorest Billion](http://ncdsynergies.org/)^51^
2. Webpage (for browsing): [CDC Global Noncommunicable Diseases (NCDs)](https://www.cdc.gov/globalhealth/healthprotection/ncd/index.html)^52^
3. Webpage (for browsing): [WHO: Noncommunicable Diseases](https://www.who.int/news-room/fact-sheets/detail/noncommunicable-diseases)^53^
4. Article: [Healthy people and healthy profits? Elaborating a conceptual framework for governing the commercial determinants of non-communicable diseases and identifying options for reducing risk exposure](https://www.ncbi.nlm.nih.gov/pmc/articles/PMC5472958/)^54^
5. Optional resource: [The PIH NCD guidebook](https://www.pih.org/practitioner-resource/the-pih-guide-to-chronic-care-integration-for-endemic-non-communicable-dise)^55^

Example Speakers

1. [Paul H. Park, MD, MSc](https://ghsm.hms.harvard.edu/faculty-staff/paul-h-park)is the Director of Implementation for NCD Synergies at Partners In Health and holds appointments in the Department of Global Health and Social Medicine at Harvard Medical School, the Division of Global Health Equity at Brigham and Women’s Hospital, and the University of Global Health Equity in Rwanda
2. [Gene F. Kwan, MD, MPH](https://www.bumc.bu.edu/busm/profile/gene-kwan/) is a cardiologist and global health researcher developing expertise in the intersection between those two fields
3. [Gene Bukhman, MD, PhD](https://ghsm.hms.harvard.edu/faculty-staff/gene-bukhman) is a cardiologist and medical anthropologist who heads the Program in Global Noncommunicable Disease and Social Change at Harvard Medical School

# To Sustain, or Not to Sustain…

Session Description

There currently exist massive global processes that many see as a threat to our modern civilization. These include climate change, uncontrolled population growth fueled by extreme poverty, the pandemic diseases such as Ebola or COVID-19, and the risk of global financial collapse (as was seen in 2008 when “the housing bubble” popped). What needs to be done to address these issues adequately? Is it currently being done? Or what else do we as a human species need to do? Who needs to do it? And what is that work in the first place? This session puts trainees in contact with anyone who is being thoughtful about these questions. There will be no easy answers but asking the right questions in the first step.

Profile of speaker: Potential speakers could be an academician, a reporter, or a public health professional who has covered or studied climate change, pandemics, or extreme poverty. The goal is not to cover one agenda over another or to win converts to one political agenda over another. Instead, the goal is to engage in a thoughtful conversation that sheds light on these realities and gets students thinking about their potential role in this new future.

Questions for Facilitation

*Opening narrative: Many of the biggest challenges facing global health simply don't need to be so hard. They come from the creation of systems that favor what is easiest: consumption that leads to unprecedented pollution and climate change, aid policies that simply aren't enough and favor the wrong players, inequality that leads to insecurity for the poor which leads to unsustainable population growth which, in turn, leaves billions vulnerably hanging over the pit of the next plague, etc. Calamity falls upon calamity. What will it take to create real change for a sustainable future? The Brundtland Report on "Our Common Future" published in 1987 painted a different way, and Greta Thunberg made an impassioned plea for similar themes at the 2019 UN General assembly, but we're still not hearing the message. In this session, we're going to sit and talk about what the sharp end of an unsustainable future looks like. This will hopefully lead us to new insights about how to get the world onto a better “common future."*

- *To the discussant*: What concerns you the most about the future of modern civilization? And what gives you the most optimism about the future of civilization?
- *To the group*: Are we doing enough to confront the biggest problems facing our planet? If so, what are we doing well? If not, what else can we do?
- *If the discussant describes the transformative role of technology, consider these questions:*
  - What do you think is the most promising technology to confront these issues?
  - One of the issues that we have seen with technology is that while new technology may be revolutionary, the dissemination and uptake of new technology often follows patterns of inequity; it historically has not been distributed to those who might need it the most. What do you think about this challenge with adequately disseminating promising innovations?
- *If the discussant talks about the transformative role of government, consider these questions:*
  - Some see government as the solution, others see it as the problem. This dinner is not to discuss who is right, but rather to see the merits of both sides of the argument. What is the power of the government to support massive change? What are the dangers of this power? What is the role of the market and the private sector in sparking change? Are there examples where this has worked?
- *If the discussant talks about the transformative role of social movements, consider these questions:*
  - What have different social movements done in the past that made them so successful?
  - Examples of successful social movements might include civil rights, apartheid, etc., whereas examples of failed social movements include the prohibition of alcohol. Other movements are currently ongoing (e.g., black lives matter). Based on your experiences, can you reflect on why some movements succeed and some fail?
- *If the discussant talks about the transformative power of anything else (X), consider these questions:*
  - What led you to see X as a game-changer?
  - Do you see any unintended consequences of X?
  - What can be done to mitigate the unintended consequences of X?
  - What can we do to accelerate the progress of X?
  - What are the biggest factors slowing down progress via X?

Student/Resident Preparation

1. Article: [Sustainable Humanity - Jeffrey D. Sachs](https://www.earth.columbia.edu/sitefiles/file/Sachs%20Writing/2012/ProjectSyndicate_2012_SustainableHumanity_01_31_12.pdf)^56^
2. Article: [World to Poor: Drop Dead](https://www.huffpost.com/entry/world-to-poor-drop-dead_b_4385420)^57^
3. Video: [Lola Dare and Anders Nordström at Global Health Beyond 2015](https://www.youtube.com/watch?v=-ol9eu2NivM&feature=youtu.be)^58^ (32 minutes)
4. Article: [The Brundtland Report: Report of the World Commission on Environment and Development: Our Common Future](http://www.un-documents.net/wced-ocf.htm)^59^

Example Speakers

1. [Dan Kelly, MD, MPH](https://profiles.ucsf.edu/john.d.kelly#toc-id5) is an infectious diseases physician at UCSF. He continues to conduct Ebola research in Sierra Leon and Liberia. He employs sero-epidemiology, transmission mapping and modeling techniques, and clinical research cohorts to investigate asymptomatic Ebola virus infection and mild illness.

SUPPLEMENTAL CURRICULUM

*Global health encompasses many fields and concentrations. The supplemental curriculum consists of deep dives into a few of global health’s many sub-concentrations. Given the specificity of the topics, these dinners may present unique challenges when attempting to secure a knowledgeable speaker with relevant experience. However, this supplemental curriculum can be a fruitful addition to the core curriculum and will serve to broaden trainees’ understanding of global health.*

# Health Care Delivery in Post-Genocide States

Session Description

Countless different episodes of genocide have unfortunately occurred, but many of them follow a stereotypical pattern: conflict arises between two groups that are socially stratified; they are often in disagreement because of a scarce resource, so one group works to dehumanize the other group, often by comparing them to non-human things (vermin, insects, etc.); this is then followed by a concerted effort to kill. Indeed, genocides are marked by their common convergence on this path of death, but once the killing has stopped different post-conflict societies have subsequently taken very different pathways. Some societies such as Rwanda have entered into a deep and difficult dialogue for reconciliation. Other societies such as Guatemala have only been thrust deeper into poverty and conflict despite the signing of peace accords.

Profile of speaker: This dinner will ideally put trainees in contact with someone who has experience working in a society affected by genocide. This could be an aid worker or military personnel who worked in an area affected by genocide, or a person who fled genocide. Ideally, if there are two people who have worked or fled from two different genocides, hosting both of them at one dinner may offer unique insights into why genocides occur, how they can be prevented, and how different societies heal from the trauma.

Questions for Facilitation

*Opening narrative: The goal of this discussion will not be to simply compare different post-genocide societies, nor to contrast their tragedies. Instead, we will explore the universal lessons that may be available to us if we later find ourselves in global health work that asks us to negotiate the sins of history, the uncertainties of the past, and the possibilities of the future.*

- *To the discussant*: Thank you for agreeing to meet with us. We can walk about as much or as little as you are willing to discuss, as we are aware that these can be difficult conversations. With that in mind, can you describe to us what happened in your country/the country where you worked?
- *To the discussant*: Were you personally affected by the genocide?
- *To the group*: The slogan often raised after each genocide is "never again," but yet, history repeats itself too often. This was stated uniquely in a famous poem from Gertrud Stein when she said: "Let us learn what history teaches. History teaches." In your opinion, are we doomed to repeat tragedies such as this? Or what will need to be done to assure genocides truly never again happen, and we finally learn from history?
- *To the discussant*: What is it like to provide healthcare in a post-genocide state? What are the specific system challenges and medical needs?
- *To the discussant*: A big part of a society healing itself after genocide is reconciliation. What is the role of healthcare providers in the reconciliation process? What works well? And what does not work?

Student/Resident Resources

1. Podcast: [This American Life Episode #465: What Happened At Dos Erres](https://www.thisamericanlife.org/465/what-happened-at-dos-erres)^60^ (59 minutes)
2. Podcast: [This American Life Episode #683: Beer Summit (Prologue and Act One only)](https://www.thisamericanlife.org/683/beer-summit)^61^ (40 minutes)
3. Website (for browsing): [World Without Genocide](http://worldwithoutgenocide.org/genocides-and-conflicts/rwandan-genocide)^62^
4. (Optional) Movie: [Hotel Rwanda (2004)](https://en.wikipedia.org/wiki/Hotel_Rwanda)^63^

Example Speakers

1. [Peter J. Rohloff, MD, Ph.D.](https://connects.catalyst.harvard.edu/Profiles/display/Person/61589) is an assistant professor of medicine at Brigham and Women’s Hospital and the co-founder and Chief Medical Officer for Maya Health Alliance, a primary care organization working with indigenous Maya communities in rural Guatemala.
2. [Emmanuel Kamanzi, MBA](https://ughe.org/meet-the-team/emmanuel-kamanzi/) is the Director of Infrastructure at the University of Global Health Equity (UGHE). He Managed UGHE’s 100,0000 Square feet inaugural Campus of Butaro and he is in charge of the planning, development and maintenance of all University Infrastructure.

# The Medical Implications of the Second Amendment

Session Description

Barack Obama's nomination for surgeon general, Vivek Murthy, was met with significant opposition from the Republican Party and the National Rifle Association (NRA) because he called gun violence a public health issue. The opposition came from the NRA because they were concerned that he was going to use his medical position to influence how the second amendment is interpreted. Later on, the NRA demanded on Twitter that physicians to "stay in their lane" when thinking to study or discuss the health effects of gun reform, to which multiple physicians responded, “This is our f***ing lane.” The current situation in America is such that gun violence will affect many of our lives and all of our practices, but our current political processes are woefully inadequate to offer effective solutions. What role does the physician have, therefore, in this reality? What responsibility does the physician have in the face of this tragedy? What has worked in other countries? And what has failed?

Profile of speaker: A healthcare provider who works in trauma (e.g., a trauma surgeon who cares for victims of gun violence) and/or someone who works in policy reform for gun violence, whether at the local, state, or national levels.

Questions for Facilitation

*Opening narrative: This session serves not only to draw attention to the national issue of gun violence but also to demonstrate how physicians can use their training and authority to address issues in their local or national community. This is a heavily politicized issue but our goal here is not to debate the politics; instead, let’s focus on how we can keep our patients safe.*

- *To the discussant*: Can you describe your understanding of the problem with gun violence in America?
- *To the discussant*: What works to curb gun violence? Some say give people more guns, and teach them how to use them better; others say to limit access to guns. What does the evidence show works best?
- *To the discussant*: Of all the options, what is most feasible in the US context?
- *To the group*: Australia's experience with gun reform is that limiting access to guns decreased gun violence. Is this solution possible in the US?
- *To the discussant*: How does gun violence affect your everyday practice? Can you tell us a story that encapsulates your experience with gun violence, or that captures your current understanding about gun violence?
- *To the group*: Some point out that gun violence is actually a problem of something other than guns, e.g., poverty or mental health issues. The NRA has coined statements such as “guns don’t kill people; people kill people” and “the only thing that can stop a bad guy with a gun is a good guy with a gun,” and these have been very influential in influencing public opinion. How do you all interpret this debate?
- *To the group*: If you each had a magic wand and could enact the perfect gun safety policy, how would that look like?

Student/Resident Preparation

1. Article: [Congress Quashed Research Into Gun Violence. Since Then, 600,000 People Have Been Shot.](https://www.nytimes.com/2018/03/12/health/gun-violence-research-cdc.html)^64^
2. Podcast: [Doctors Take On Gun Violence, NRA Pushes Back: A New Battle Over Policy.](https://www.wbur.org/onpoint/2018/11/16/doctors-nra-gun-violence-stay-in-your-lane)^65^ (47 minutes)
3. Article: [What Should Be the Scope of Physicians’ Roles in Responding to Gun Violence?](https://journalofethics.ama-assn.org/article/what-should-be-scope-physicians-roles-responding-gun-violence/2018-01)^66^
4. Article: [#ThisIsOurLane — Firearm Safety as Health Care’s Highway](https://www.nejm.org/doi/10.1056/NEJMp1815462)^67^

Example Speakers

1. [Mark E. Cichon, DO, FACEP, FACOEP](https://www.loyolamedicine.org/doctor/mark-cichon) served as Medical Director of Emergency Department and Aeromedical Transport, Loyola University Medical Center at Loyola University Health System. He chairs the Region 8 Emergency Medical Services Advisory Committee, is a member of the Illinois Medical Emergency Response Team and is a bioterrorism/mass casualty consultant for the Cook County Department of Public Health.
2. [Eric Fleegler, MD, MPH](http://www.childrenshospital.org/research/researchers/f/eric-fleegler) is a pediatric emergency physician and health services researcher at Boston Children’s Hospital. He has 14 years’ experience leading large-scale research projects and performing injury prevention research with a focus on violence and firearm fatalities.

# Trans Lives & Healthcare

Session Description

There exists a growing awareness that the trans community has unique healthcare needs that are not being adequately addressed by the healthcare system. This is because of many reasons, but very important among them is that trans patients feel unwelcome, unsafe, and misunderstood by the medical community. How can we change our practices in order to be more receptive and engaging with this community?

Profile of speaker: Someone who has experience being a provider for trans people (e.g., a member of a specialty clinic or a surgeon) or someone who has done activist work with and for the trans community. It is particularly powerful if this clinician can be paired with one of their trans patients who is comfortable with speaking about their experiences and educating the medical community.

Questions for Facilitation

*Opening narrative: Imagine that you're taking care of a patient that you have never taken care of before. You might not understand their disease, and you might not understand their experience as human beings. Their life experience is so different from yours that when you approach them, initially, there is a huge barrier between what you have experienced and what they have experienced. That might sound farcical, but that happens every day when physicians take care of trans patients. For the physician, this is may be an uncomfortable experience. For the patient, this is even worse.*

- *To the discussant(s)*: What do you recommend the physician should know before taking care of trans patients?
- *To the discussant(s)*: [Clinician], can you tell us about your experiences caring for trans patients? [Patient], can you tell us about your experiences as a trans patient?
- *To the group*: Give us an example of a really good (and then a really bad) care experience you've seen with trans patients. What are the lessons we can learn from these experiences?
- *To the discussant(s)*: One of the core concerns that trans patients often express is that clinicians, not just physicians, have an undue curiosity about their bodies. How can we adapt our history and physical so as to be sensitive to this concern? Are there other concerns of which we should be aware?
- *To the discussant(s)*: Similarly, what are other system-wide changes you would recommend to the medical system to better care for trans patients?
- *To the discussant(s)*: What are the societal changes that may need to occur for our trans patients to lead safer lives, and achieve greater levels of health and wellness?

Student/Resident Preparation

1. Podcast: [The Curbsiders Episode #72: Transgender Care in Primary Care](https://thecurbsiders.com/podcast/72-transgender-care-primary-care)^68^ (1 hour)
2. Article: [Health Care Disparities Among Lesbian, Gay, Bisexual, and Transgender Youth: A Literature Review](https://www.ncbi.nlm.nih.gov/pmc/articles/PMC5478215/)^69^
3. Article: [Care of Transgender Persons](https://www.nejm.org/doi/full/10.1056/NEJMcp1903650?query=TOC)^70^

Example Speakers

1. [Robert Garofalo, MD, MPH](https://www.luriechildrens.org/en/doctors/garofalo-robert-1632/) is the Division Head of Adolescent and Young Adult Medicine and a Professor of Pediatrics at Northwestern University Feinberg School of Medicine. His primary clinical and research activities relate to the care of marginalized youth populations including HIV+ and LGBT young people. His research is largely HIV prevention in nature, mostly targeting either young men who have sex with men or transgender individuals.

# You Are on Indigenous Land

Session Description

All of America was once indigenous land, but now only small plots remain in the form of reservations. Some, such as the Cheyenne, were forced to leave their native lands to now inhabit a very different landscape. The trauma of this formed migration reverberates to this day. Other indigenous groups, such as the Navajo, were able to stay on parts of their original land, but they too suffered historic traumas that have repercussions to this day. What do these injustices represent for indigenous people, and what should they represent for non-indigenous Americans who may care but yet have benefited tremendously from the economic growth made possible by this land? What can we learn from this history, and where do we go from here? This session will sensitize the students to this history, but also provide some useful skills for both caring for native Americans and potentially participating in advocacy that hopes to address the social determinants.

Profile of speaker: Someone who has worked with the Indian Health Services (IHS) or a representative from a native community. If possible, someone who worked as a community health representative (a type of community health worker) in the IHS is often a great discussant because they bridge the gap between the communities and the healthcare systems hoping to serve them.

Questions for Facilitation

*Opening narrative: Indigenous populations suffer some of the worse health outcomes in the US. This is because of historical injustices that have been deeply encoded into the social determinants of disease that continue to affect them even to this day. What role can the healthcare provider play in this reality? What responsibility does the healthcare provider in America have to correct these injustices?*

- *To the discussant:*
  - Tell us about your experiences working with indigenous populations in the US?
- *To the speaker(s), Native American or non-native*:
  - From your perspective, what are some of the most important health-related needs that Native Americans face?
  - In your opinion, what are the best ways that the US healthcare system or IHS can address these?
  - What are policies on either a district, state, or national level that need to be put into place to substantially change the health and wellbeing of native populations?
  - Are there initiatives or movements that we should know about, or participate in, to support these efforts?
  - How can a non-native person be most helpful? How can we avoid not being helpful? For example, can you give me an example of when a non-native ally was helpful? Can you give me an example of when a non-native ally was not helpful?
- *To the group*:
  - How do we process this tragic history? What do we do with the thought that much of our country’s wealth is from land that was stolen?

Student/Resident Preparation

1. Website (for browsing): [Native Land Map](https://native-land.ca/)^71^
2. Documentary: [The Return of Navajo Boy](https://navajoboy.com/)^72^ (58 minutes)
3. Documentary Series: [We Shall Remain Collection](https://www.pbs.org/wgbh/americanexperience/films/weshallremain/)^73^

Example Speakers

1. Sherry Begaye, Larissa Morgan, and Brenette Pine, all Community Health Representatives from the Diné (Navajo) Nation and that have collaborated with the [Community Outreach and Patient Empowerment Program](https://www.copeprogram.org/) of the Navajo Nation

# The Opioid Crisis: How Did We Get Here and Where Do We Go?

Session Description

For many physicians currently in clinical practice, they can remember a time when they were paged by nurses saying that "the patient is in pain and pain is the 5th vital sign. Please order something." The common practice at the time was to prescribe opiates first. This was largely because physicians at that time were taught that the short-term use of opiates in cases of acute pain did not increase rates of addiction. For many patients, however, this short exposure to opiates led them down the path to addiction. Simultaneously, many patients were being maintained on high doses of narcotics for chronic pain. These scripts flooded American homes with opiates, and many friends and family members of those patients tried their first opiate by sampling that excess supply. Why physicians believed that this was the right thing to do is a more complex story that is still being untangled, but the strings are leading back to pharmaceutical corporate greed, willful manipulation, and broader socio-economic forces that made opiates a band-aid solution for much more pervasive social suffering.

Profile of speaker: Anyone who works in addiction medicine, an administrator at a specialty addiction recovery clinic, a public health researcher who has investigated the opioid crisis, and/or a politician or lawyer who works on opioid addiction-related policies.

Questions for Facilitation

*Opening narrative: The opioid crisis is a big problem with iatrogenic roots. Since doctors partially created this problem, how can we aid in its correction? The ultimate goal of tonight is to leave here with a sense of the best way to approach patients with opioid use disorder and where we can access the latest resources for its prevention and treatment. We will also work to contextualize this problem within the larger social and historical forces that helped shape it in the first place.*

- *To the discussant:* Can you tell us your understanding of why America is in the throes of an opioid addiction crisis?
- *To the discussant:* Why is the addiction crisis worse in some areas over others?
- *To the discussant:* What medical technologies do we currently have available to us to confront this crisis?
- *To the discussant:* Can you tell us your practical experience in confronting the crisis, such as your experiences with patients, or any experiences you had working to support and/or enact policy change?
- *To the discussant:* In your opinion, what are the policy changes that need to occur to better confront this crisis?
- *To the group:* Do you think that we will get over this crisis? What role can you play in this work?
- *To the discussant:* Not everyone will work in advocacy or policy around these issues, but everyone will be affected by addiction in their practice. What advice would you give all practitioners in light of this new reality?

Student/Resident Preparation

1. Video: [TEDx: Addiction is a Disease. We Should treat it like one.](https://www.ted.com/talks/michael_botticelli_addiction_is_a_disease_we_should_treat_it_like_one?language=en)^74^ (11 minutes)
2. Article: [How a Police Chief, a Governor and a Sociologist would Spend $100 Billion to Solve the Opioid Crisis](https://www.nytimes.com/interactive/2018/02/14/upshot/opioid-crisis-solutions.html)^75^
3. Article: [Opioid Crisis: No Easy Fix to its Social and Economic Determinants](https://ajph.aphapublications.org/doi/10.2105/AJPH.2017.304187)^76^
4. Article: [The Opioid Diaries](https://time.com/james-nachtwey-opioid-addiction-america/)^77^
5. Article: [When an Iowa Family Doctor Takes on the Opioid Epidemic](https://www.nytimes.com/2018/06/23/health/opioid-addiction-suboxone-treatment.html)^78^
6. Podcast: [The American Health Podcast: Treating Opioid Addiction](http://americanhealth.libsyn.com/treating-opioid-addiction)^79^ (29 minutes)
7. Video: [TEDx: Addiction 101](https://www.youtube.com/watch?v=S86t89HOYlE&app=desktop)^80^ (14 minutes)

Example Speakers

1. [Elizabeth Salisbury-Afshar MD, MPH](https://www.air.org/person/elizabeth-salisbury-afshar) is a board-certified family physician and the director of the Center for Addiction REearch and Effective Solutions at the American Institutes for Research. With experience working on opioid-related epidemiology, policy, public health intervention/evaluation, and serving directly as a treatment provider, she leads the Center’s work in research, policy and practice.
2. [Nicole Gastala, MD](https://hospital.uillinois.edu/find-a-doctor/nicole-gastala) practices in the Department of Family Medicine at UI Health. Her work focuses on preventative healthcare and chronic disease management for pediatric, adolescent, adult, and OB/GYN patients. She has an interest in addiction medicine, including medication-assisted treatment for opioid-use disorder.

# Human Trafficking

Session Description

Survivors of human trafficking will likely not provide that information without prompting, and many providers do not include human trafficking screening as part of their social history. How then can we identify and care for this population? What are the resources that physicians can offer these patients, and how can we use our medical skillset best for this vulnerable population?

Profile of speaker: Anyone who works in trafficking reduction through policy and/or is a healthcare worker whose practice is to support children and/or adults who have been traumatized by trafficking.

Questions for Facilitation

*Opening narrative: Individuals who experience human trafficking have a range of health consequences, and many individuals with pervasive health concerns and disabilities are particularly vulnerable to traffickers. Even though many trafficking victims actually access health care during their trafficking situation, not every health care professional is equipped to identify the signs, or how to address a trafficking survivor's needs.*

- *To the discussant:* Can you describe the problem of human trafficking as you understand it?
- *To the discussant:* Can you describe your work in combating human trafficking?
- *To the group:* For many people, human trafficking is not even on their radar, which goes to show that much of the suffering goes on in silence. How do you think we should help break that silence?
- *To the discussant:* What are the unique medical needs of this patient population? What are the legal needs, and how does a physician with little experience in medical-legal partnerships proceed when they find one of their patients is being trafficked?
- *To the discussant:* How is it possible that our legal and societal systems are failing these kids?
- *To the discussant:* If someone wants to dedicate their professional careers to this issue, what advice would you give them?
- *To the discussant:* If someone does not want to dedicate their professional careers to this issue but wants to be an ally, what advice would you give them?

Student/Resident Preparation

1. Article: [Multi-level prevention of human trafficking: The role of health care professionals](https://www.sciencedirect.com/science/article/pii/S0091743518302135)^81^
2. Video: [Look Beneath the Surface](https://www.youtube.com/watch?v=l76cqmHI_k0&feature=youtu.be)^82^ (14 minutes)

Example Speakers

1. Cassandra Ma, PsyD is the Executive Director of [Reclaim13](http://www.reclaim13.org/) an NGO which seeks to support victims of trafficking and to break the cycle of child sex trafficking in the United States

# Incarcerated But Still Human: The Practice of Correctional Healthcare

Session Description

Patients who have passed through the correctional system are known to have an increased risk of infectious and noncommunicable diseases while, at the same time, they are unlikely to receive regular medical care. For some, physician visits within the correctional system may be the only medical care they can access in the period before, during, and after their incarceration. The specific niche of correctional healthcare is often not discussed in medical school or residency programs, so most physicians do not know how to best care for this unique population.

Profile of speaker: A healthcare worker that works, or has worked, in a correctional facility or an administrator that oversees the provision of healthcare to incarcerated persons.

Questions for Facilitation

*Opening narrative: Inmates in correctional facilities have significantly higher rates of disease than the general population. This population tends to suffer in greater numbers from infectious disease, mental health problems, and substance use and addiction. What does healthcare look like for incarcerated persons? How do inmates continue their care once they are released? These questions and others will be answered during this session as we try to get a better understanding of the not-often-discussed correctional healthcare system which provides healthcare to millions of Americans.*

- *To the discussant:* Can you tell us about your work taking care of incarcerated patients?
- *To the discussant:* Can you discuss some of the challenges?
- *To the discussant:* Why did you choose to do this type of work?
- *To the discussant:* If someone wanted to pursue this kind of work, what advice would you give them?
- *To the discussant:* I imagine you see many difficult and heartbreaking stories each day. How do you do this kind of work without becoming overwhelmed by the stories you encounter each day?
- *To the discussant:* How do you provide continuity of care once people leave jail?
- *To the discussant:* What can be done to help patients engage with care long-term?
- *To the discussant:* Critics of the US prison system sometimes describe our current levels of incarceration of young African American men as a new form of Jim Crow. What are your thoughts about that? What are possible solutions to address this issue?
- *To the group:* As of 2020, the US has the largest population in prison in the world, and the majority are African American, and African Americans are disproportionately represented in prison populations compared to their representation in society. Many are there for non-violent offenses (such as selling Marijuana, even though Marijuana is now being legalized in many states across the US). The physician that cares for prisoners is one of the most proximate professionals to this reality. Is the system unjust? If so, what responsibility does the physician have in the face of that injustice?

Student/Resident Preparation

1. Article: [Medicine and the Epidemic of Incarceration in the United States](https://www.ncbi.nlm.nih.gov/pmc/articles/PMC3154686/)^83^
2. Article: [HIV Care in the Correctional Setting: An Overview of the Reality and the Challenges](https://www.thebodypro.com/article/hiv-care-correctional-setting)^84^
3. Podcast: [Ethics Talk: What Are Clinicians’ Responsibilities to Incarcerated Patients?](https://journalofethics.ama-assn.org/podcast/ethics-talk-what-are-clinicians-responsibilities-incarcerated-patients)^85^ (23 minutes)

Example Speakers

1. [Chad Jeremy Zawitz, MD](https://www.rushu.rush.edu/faculty/chad-j-zawitz-md) is an infectious disease specialist at Cermak Health Services and senior physician/clinical coordinator of HIV and infectious disease services with additional privileges at John H. Stroger Hospital and CORE Center, Cermak physician chair of Infection Control/Communicable Diseases Department, the Cermak director of both Infectious Diseases and the Tuberculosis Screening and Treatment Program.

# The US Healthcare System: A Look at Possible Solutions

Session Description

Although the experience with the passage of the Affordable Care Act, also known as “Obamacare,” for most Americans conjures up images of political stalemate and difficult conversations over Thanksgiving dinner, we continue to be in a moment of great opportunity and innovation in American medicine. Beyond the traditional opportunities of innovations in technology and pharmaceuticals, there are now also opportunities to innovate in care delivery mechanisms, especially with a focus on quality and equity. This session will expose students to the innovators who learned important lessons in the post-Obamacare context. Also important to represent are the voices of dissent, those that resist government-led healthcare reform and would prefer another route.

Profile of speaker: Someone who has worked in healthcare policy reform on any level (federal/state/local government, health system, or hospital levels), someone with private sector experience in healthcare administration (a CEO or CMO of a health system or a high-level administrator of a health insurance company) , an active member of a national healthcare reform organization (e.g., “Physicians for a National Health Plan” or “Doctors for America”) and/or someone with a background in healthcare economics or policy.

Questions for Facilitation

*Opening narrative: The ACA was a market-based solution that attempted to nudge our healthcare system towards greater accountability for outcomes with the hopes that this would decrease costs and increase quality. This happened most successfully in the states where the program was implemented as designed, which included an expansion of Medicaid for the poorest and sickest. In the states where this happened, such as Massachusetts, universal health coverage was achieved and health expenditures for the poor increased. In states where this did not happen, the high-risk patients stayed in the insurance marketplace and their increased expenditures drove up premiums for all others. Obamacare started as a Republican plan, but ironically the greatest risk to it going forward are Republican governors. At the same time, some Republican governors, such as Mike Pence in Indiana, have rolled out Medicare on very different terms with some success. Some Accountable Care Organizations have innovated with new ways of keeping populations healthy. Regardless of what is next, this historic experiment in healthcare reform holds countless opportunities to learn about how our health system delivers care to whom and how well.*

- *To the group*:
  - On a light note, what were you doing the day you heard Obamacare passed? How did you feel? (Go around, tell the stories)
  - What where you doing the day you heard Trump was elected? How did you feel? (Go around, tell the stories)
- *To the discussant*:
  - Tell us about your work.
  - What opportunities did you see begin or end because of the passage of the Affordable Care act?
  - What do you see as important next steps in innovation for the American healthcare system?
  - If you had a magic wand, what would you do to the US healthcare system to improve it?
  - What do you think is coming next in healthcare reform?
  - Are there some things that are just good practice in care delivery outside of politics? (e.g., is universal access to vaccinations indispensable?). If so, can we protect those things from the political waves of reform, repeal, and replace?
  - No one can predict the future, but looking into the distance, what advice would you give young clinicians who are now only staring their careers?

Student/Resident Preparation

1. Podcast: [WGN Radio: Dr. David Ansell](https://wgnradio.com/2017/10/04/dr-david-ansell-four-more-stop-son-the-blue-line-and-life-expectancy-plummets/)^86^ (13 minutes)
2. Podcast: [Freakonomics: How Do We Really Know What Works In Healthcare?](http://freakonomics.com/podcast/how-do-we-know-what-really-works-in-healthcare-a-new-freakonomics-radio-podcast/)^87^ (43 minutes)
3. Article: [JAMA: To Isaiah](https://www.bu.edu/familymed/files/2016/08/Isaiah_Berwick_JAMA2012.pdf)^88^
4. Optional, but recommended article: [The Anatomy of Healthcare in the United States](https://commed.vcu.edu/IntroPH/Introduction/2014/Thersocalledsysterm.pdf)^89^

Example Speakers

1. [David A. Ansell, MD, MPH](https://www.rush.edu/about-us/about-rush/leadership/david-ansell-md-mph-about-rush) is the Senior Vice President for Community Health Equity and the Associate Provost for Community Affairs at Rush University. He is leading Rush’s strategy to be a catalyst for community health and economic vitality on Chicago’s West Side.
2. [Andrew L. Ellner, MD](https://primarycare.hms.harvard.edu/faculty-staff/andrew-ellner) is a faculty affiliate and the founding co-director of the Center for Primary Care at Harvard Medical School.
3. [David Matheson, JD, MBA](https://www.wittkieffer.com/consultant/dave-matheson/) is a senior advisor to The Boston Consulting Group, where he worked for 30 years, serving as a senior partner and in various other capacities.

References

1. Glass I. Episode 364: Going Big -  Act One: Harlem Renaissance. *This American Life.* Published September 26, 2008.

2. Palazuelos D, Dhillon R. Addressing the “global health tax” and “wild cards”: practical challenges to building academic careers in global health. *Academic Medicine.* 2016;91(1):30.

3. Glass I. Episode 391: More Is Less. *This American Life.* Published October 9, 2009.

4. Glass I. Episode 392: Someone Else's Money. *This American Life.* Published October 16, 2009.

5. Hiatt HH. Medical Lifeboat: Will There Be Room for You in the Health Care System? 1989.

6. Glass I. Episode 403: NUUMMI. *This American Life.* Published March 26, 2010.

7. Glass I. Episode 431: See No Evil - Act Three: I Worked at the Kennedy Center and All I Got Was This Lousy T-Shirt. *This American Life.* Published April 1, 2011.

8. Schwarz R, Maru DS-R, Schwarz D, et al. On Partnership. *Narrative inquiry in bioethics.* 2012;2(2):101-106.

9. Riviello ED. Internship in Africa: Death and life. *Annals of internal medicine.* 2008;149(5):353-354.

10. Glass I. Episode 491: Tribes. *This American Life.* Published March 29, 2013.

11. Westerhaus M, Finnegan A, Goldsmith J, Lyon E, Fox C, Morse M. Social justice should be a key part of educating health professionals. *STAT News.* Published April 7, 2017.

12. Nordell J. Is this how discrimination ends. *The Atlantic.* 2017;7.

13. Bloom P. The original colonists. *The New York Times Book Review.* 2012:30-31.

14. Kim J. World Bank Group President Jim Yong Kim's Speech at World Health Assembly: Poverty, Health and the Human Future. *Retrieved.* 2013;1(17):2014.

15. Aizenman N. Scientists Say It's Time To End 'Parachute Research'. *NPR: Goats and Soda.*

16. Shah SK, Nodell B, Montano SM, Behrens C, Zunt JR. Clinical research and global health: mentoring the next generation of health care students. *Global public health.* 2011;6(3):234-246.

17. Madon T, Hofman KJ, Kupfer L, Glass RI. Implementation science. In: American Association for the Advancement of Science; 2007.

18. Hammitt LL, Akech DO, Morpeth SC, et al. Population effect of 10-valent pneumococcal conjugate vaccine on nasopharyngeal carriage of Streptococcus pneumoniae and non-typeable Haemophilus influenzae in Kilifi, Kenya: findings from cross-sectional carriage studies. *The Lancet Global Health.* 2014;2(7):e397-e405.

19. National Institutes of Health. NIH Tips for Applicants. In: June 16, 2010:<https://www.youtube.com/watch?v=9cNRMsCGfHo&feature=youtu.be>.

20. The Lancet. Eliminating TB. In:October 26, 2015:<https://www.thelancet.com/doi/story/10.1016/audio.2015.1010.1023.2404>.

21. Earnest MA, Wong SL, Federico SG. Perspective: physician advocacy: what is it and how do we do it? *Academic medicine.* 2010;85(1):63-67.

22. Luft LM. The essential role of physician as advocate: how and why we pass it on. *Canadian medical education journal.* 2017;8(3):e109.

23. Keshavjee S. *Blind spot: how neoliberalism infiltrated global health.* Vol 30: Univ of California Press; 2014.

24. van Dernoot Lipsky L. Beyond the Cliff |  TEDx Washington Corrections Center for Women. In:April 23, 2015:<https://www.youtube.com/watch?v=uOzDGrcvmus&feature=emb_title>.

25. Toy S. 45 years ago, the nation learned about the Tuskegee Syphilis Study. Its repercussions are still felt today. *USA Today.*

26. ACES Too High News. Got Your ACE Score? <https://acestoohigh.com/got-your-ace-score/>. Accessed2019.

27. Blakemore E. The Little-Known History of the Forced Sterilization of Native American Women. In: JSTOR; 2016.

28. Withers M. Trauma-Informed Care and Why It Matters. *Psychology Today.* Published July 6, 2017.

29. Rajani N. Season 2 Episode 8: The hidden costs of war. *Médecins Sans Frontières Podcast.* Published July 14, 2017.

30. Center for Strategic & International Studies. The New Barbarianism. In:September 26, 2017:<https://www.csis.org/features/new-barbarianism>.

31. Sahloul Z. What can we learn from the worst humanitarian crisis of our time? | TEDx U of I Chicago. In:July 2, 2018:<https://www.youtube.com/watch?v=ISVxZZX1BDY>.

32. Fawal J. The 5 types of mentors you need in your life. *IDEASTEDCOM.* Published September 18, 2018.

33. Tjan T. Why Leadership and Mentorship Does Not Need to Fail Us | TEDx Beacon Street. In:February 23, 2018:<https://www.youtube.com/watch?v=mibnG8XeHlU&feature=emb_title>.

34. Palazuelos D, Dhillon R, Nelson AK, et al. Training toward a movement: career development insights from the first 7 years of a global health equity residency. *Journal of Graduate Medical Education.* 2018;10(5):509-516.

35. Manchanda R. What makes us sick? Look upstream | TEDSalon NY 2014. In:August 2014:<https://www.ted.com/talks/rishi_manchanda_what_makes_us_get_sick_look_upstream?utm_campaign=tedspread&utm_medium=referral&utm_source=tedcomshare>.

36. Metzl JM, Hansen H. Structural competency: Theorizing a new medical engagement with stigma and inequality. *Social science & medicine.* 2014;103:126-133.

37. Carrasco H, Messac L, Holmes SM. Misrecognition and Critical Consciousness-An 18-Month-Old Boy with Pneumonia and Chronic Malnutrition. *The New England journal of medicine.* 2019;380(25):2385-2389.

38. Steger K. Global Health and Journalism Look for Ways to Save the World in a New Media Landscape. *Pulitzer Center.* Published September 9, 2010.

39. Panjabi R. No one should die because they live too far from a doctor | TED 2017. In:April 2017:<https://www.ted.com/talks/raj_panjabi_no_one_should_die_because_they_live_too_far_from_a_doctor>.

40. Last Mile Health. Community Health Academy. <https://lastmilehealth.org/chacademy/>. Accessed2019.

41. Community Health Impact Coalition. <https://chwimpact.org/>. Accessed2019.

42. Glass I. Episode 410: Social Contract. Published June 18, 2010.

43. Clinebell SK, Clinebell JM. The tension in business education between academic rigor and real-world relevance: The role of executive professors. *Academy of Management Learning & Education.* 2008;7(1):99-107.

44. Chin MH, Covinsky KE, McDermott MM, Thomas EJ. Building a research career in general internal medicine. *Journal of general internal medicine.* 1998;13(2):117-122.

45. Levinson W, Linzer M. What Is an academic general internist?: Career options and training pathways. *JAMA.* 2002;288(16):2045-2048.

46. Glass I. Episode 525: Call For Help. *This American Life.* Published May 9, 2014.

47. Frenk J, Moon S. Governance challenges in global health. *New England Journal of Medicine.* 2013;368(10):936-942.

48. University College London. Global Health Governance: Who is accountable to whom? *The UCL Institute for Global Health Symposium.* Published June 14, 2009.

49. Belluz J, Buissonniere M. How McKinsey infiltrated the world of global public health. *Vox.* Published December 13, 2019.

50. Noah T. Anand Giridharadas - "Winners Take All" and the Paradox of Elite Philanthropy | The Daily Show. In:October 8, 2019:<https://www.youtube.com/watch?v=H32z45o30WxA>.

51. NCD Synergies. <http://ncdsynergies.org/>. Accessed2019.

52. Centers for Disease Control and Prevention. CDC Global Noncommunicable Diseases (NCDs). <https://www.cdc.gov/globalhealth/healthprotection/ncd/index.html>. Accessed2019.

53. World Health Organization. Noncommunicable disease. <https://www.who.int/news-room/fact-sheets/detail/noncommunicable-diseases>. Accessed2019.

54. Buse K, Tanaka S, Hawkes S. Healthy people and healthy profits? Elaborating a conceptual framework for governing the commercial determinants of non-communicable diseases and identifying options for reducing risk exposure. *Globalization and health.* 2017;13(1):34.

55. Partners in Health, Bukhman G, Kidder A. *The PIH guide to chronic care integration for endemic non-communicable diseases.* Partners in Health; 2011.

56. Sachs JD. Sustainable Humanity. *Project Syndicate.* Published January 31, 2012.

57. Sachs JD. World to Poor: Drop Dead. *HuffPost.* Published February 3, 2014.

58. Dare L, Nordström A. Lola Dare and Anders Nordström at Global Health - Beyond 2015. In:April 22, 2013:<https://www.youtube.com/watch?v=-ol9eu2NivM&feature=youtu.be>.

59. Brundtland GH, Khalid M, Agnelli S, Al-Athel S, Chidzero B. Our common future. *New York.* 1987:8.

60. Glass I. Episode 465: What Happened at Dos Erres. *This American Life.* Published May 25, 2012.

61. Glass I. Episode 683: Beer Summit. *This American Life.* Published September 20, 2019.

62. World Without Genicide. Rwandan Genocide. <http://worldwithoutgenocide.org/genocides-and-conflicts/rwandan-genocide>. Accessed2019.

63. George T. Hotel Rwanda 2004.

64. Kaplan S. Congress quashed research into gun violence. Since then, 600,000 people have been shot. *New York Times.* 2018.

65. Folkenflik D. Doctors Take On Gun Violence, NRA Pushes Back: A New Battle Over Policy. *NPR: On Point.* Published November 16, 2018.

66. Jones N, Nguyen J, Strand NK, Reeves K. What Should Be the Scope of Physicians’ Roles in Responding to Gun Violence? *AMA journal of ethics.* 2018;20(1):84-90.

67. Ranney ML, Betz ME, Dark C. # ThisIsOurLane—firearm safety as health care’s highway. *New England journal of medicine.* 2019;380(5):405-407.

68. Watto M. Episode  #72: Transgender Care in Primary Care. *The Curbsiders.* Published December 11, 2017.

69. Hafeez H, Zeshan M, Tahir MA, Jahan N, Naveed S. Health care disparities among lesbian, gay, bisexual, and transgender youth: A literature review. *Cureus.* 2017;9(4).

70. Safer JD, Tangpricha V. Care of Transgender Persons. *New England Journal of Medicine.* 2019;381(25):2451-2460.

71. Native Land Digital. Native Land Map. <https://native-land.ca/>. Accessed2019.

72. Spitz J. The Return of Navajo Boy 2000.

73. Eyre C, Burns R, Nelson Jr. S, Craig D, Colt S. We Shall Remain 2009.

74. Botticelli M. Addiction is a disease: We should treat it like one | TEDxMidAtlantic. In:October 2016:<https://www.ted.com/talks/michael_botticelli_addiction_is_a_disease_we_should_treat_it_like_one?language=en>.

75. Katz J. How a police chief, a governor and a sociologist would spend $100 billion to solve the opioid crisis. *New York Times.* 2018.

76. Dasgupta N, Beletsky L, Ciccarone D. Opioid crisis: no easy fix to its social and economic determinants. *American journal of public health.* 2018;108(2):182-186.

77. Nachtwey J. The Opioid Diaries. *Time Magazine.* 2018;191(9):2018.

78. Goodnough A. When an Iowa Family Doctor Takes on the Opioid Epidemic. *The New York Times.* Published June 23, 2018.

79. Johns Hopkins Bloomberg School of Public Health. Treating Opioid Addiction. *The American Health Podcast.* Published January 10, 2018.

80. Mehta R. Addiction 101 | TEDx UofM. In:April 6, 2015:<https://www.youtube.com/watch?v=S86t89HOYlE&app=desktop>.

81. Greenbaum VJ, Titchen K, Walker-Descartes I, Feifer A, Rood CJ, Fong H-f. Multi-level prevention of human trafficking: the role of health care professionals. *Preventive medicine.* 2018;114:164-167.

82. U.S. Department of Health & Human Services - Administration for Children & Families. Look Beneath the Surface to End Trafficking. In:May 31, 2018:<https://www.youtube.com/watch?v=l76cqmHI_k70&feature=youtu.be>.

83. Rich JD, Wakeman SE, Dickman SL. Medicine and the epidemic of incarceration in the United States. *The New England journal of medicine.* 2011;364(22):2081.

84. Zawitz C. HIV care in the correctional setting. *Positively aware: the monthly journal of the Test Positive Aware Network.* 2009;20(6):30-32.

85. Thomson-DeVeaux A. Ethics Talk: What Are Clinicians’ Responsibilities to Incarcerated Patients? *AMA Journal of Ethics.* Published September 2017.

86. Williams J. Dr. David Ansell: “Four more stops on the blue line and life expectancy plummets”. In. WGN Radio Oct 4, 2017:<https://wgnradio.com/john-williams/dr-david-ansell-four-more-stop-son-the-blue-line-and-life-expectancy-plummets/>.

87. Dubner SJ. Episode 201: How Do We Really Know What Works in Healthcare? *Freakonomics Radio.* Published April 2, 2015.

88. Berwick DM. To isaiah. *JAMA.* 2012;307(24):2597-2599.

89. Moses H, Matheson DH, Dorsey ER, George BP, Sadoff D, Yoshimura S. The anatomy of health care in the United States. *Jama.* 2013;310(18):1947-1964.
