# Supplementary material for: Transformative Global Health Pedagogy: A Dinner Curriculum for Medical Students and Residents
Source: MedEdPORTAL. 2020 Dec 3;16:11044. doi: 10.15766/mep_2374-8265.11044 (PMC7727609; doi:10.15766/mep_2374-8265.11044)
Supplement: Supplementary file 1 — GH Dinner Curriculum Manual.docxPre-session Survey.docxPost-session Survey.docx [file mep_2374-8265.11044-s001.zip › B. Pre-session Survey.docx]

**Pre-Session Survey**

The purpose of this research project is to implement and evaluate our new Global Health dinner curriculum. This is a research project being conducted by _____. Your participation in this new curriculum is completely voluntary, and as part of your participation you will be asked to complete a brief questionnaire evaluating the session you attended. If you decide to participate in this research survey, you may withdraw from your participation in the curriculum at any time.

The following survey should take about 3 minutes. Your responses will be confidential, and we do not collect identifying information such as your name, email address or IP address. All data is stored in a password protected electronic format. The results of this study will be used for scholarly purposes only.

If you have any questions about the research study, please contact _____.

By completing this survey, I indicate my consent to participate in the GH dinner curriculum and survey.

Please enter your mother's first and last initials and her date of birth. This will be used as your unique identifier in order to keep the surveys anonymous. (Formatting should be FLMMDDYY. Jane Doe 12/25/65 would be entered as JD122565. Anne Smith 5/3/65 would be entered as AS050365.)

Session I'm evaluating: _____

I was able to complete all of the pre-session assignments before the dinner (not including recommended/further reading). 1= strongly disagree. 5= strongly agree.

1 2 3 4 5

What is your level of knowledge/familiarity with/ability/awareness of session objectives 1-3? 1= very much below average. 5= very much above average.

1 2 3 4 5

Please, use the space below to make any additional comments or recommendations.
