# Supplementary material for: Transformative Global Health Pedagogy: A Dinner Curriculum for Medical Students and Residents
Source: MedEdPORTAL. 2020 Dec 3;16:11044. doi: 10.15766/mep_2374-8265.11044 (PMC7727609; doi:10.15766/mep_2374-8265.11044)
Supplement: Supplementary file 1 — GH Dinner Curriculum Manual.docxPre-session Survey.docxPost-session Survey.docx [file mep_2374-8265.11044-s001.zip › C. Post-session Survey.docx]

**Post-Session Survey**

The purpose of this research project is to implement and evaluate our new Global Health dinner curriculum. This is a research project being conducted by _____. Your participation in this new curriculum is completely voluntary, and as part of your participation, you will be asked to complete a brief questionnaire evaluating the session you attended. If you decide to participate in this research survey, you may withdraw from your participation in the curriculum at any time.

The following survey should take about 5 minutes. Your responses will be confidential, and we do not collect identifying information such as your name, email address or IP address. All data is stored in a password protected electronic format. The results of this study will be used for scholarly purposes only.

If you have any questions about the research study, please contact _____.

By completing this survey, I indicate my consent to participate in the GH dinner curriculum and survey.

Please enter your mother's first and last initials and her date of birth. This will be used as your unique identifier in order to keep the surveys anonymous. (Please enter your mother's first and last initials and her date of birth. This will be used as your unique identifier in order to keep the surveys anonymous.)

Session I'm evaluating: _____

Pre-session assignments helped me adequately prepare for the sessions. 1= strongly disagree. 5= strongly agree.

1 2 3 4 5

The pre-session assignments contributed to my understanding of today's topic. 1= strongly disagree. 5= strongly agree.

1 2 3 4 5

The dinner clarified concepts I could not learn on my own. 1= strongly disagree. 5= strongly agree.

1 2 3 4 5

This session provided me with new knowledge on a global health topic. 1= strongly disagree. 5= strongly agree.

1 2 3 4 5

The guest speaker was relevant to the session topic. 1= strongly disagree. 5= strongly agree.

1 2 3 4 5

The guest speaker contributed to my learning. 1= strongly disagree. 5= strongly agree.

1 2 3 4 5

The facilitator adequately kept the flow of the dinner focused and relevant. 1= strongly disagree. 5= strongly agree.

1 2 3 4 5

This session introduced me to at least one potential mentor in Global Health. 1= strongly disagree. 5= strongly agree.

1 2 3 4 5

I prefer this dinner-style learning session over a didactic style (lecture). 1= strongly disagree. 5= strongly agree.

1 2 3 4 5

The timing of the session worked well with my schedule. 1= strongly disagree. 5= strongly agree.

1 2 3 4 5

I enjoyed the food served at the dinner. 1= strongly disagree. 5= strongly agree.

1 2 3 4 5

Your overall rating of the session. 1= Unsatisfactory. 5= excellent.

1 2 3 4 5

What is your level of knowledge/familiarity with/ability/awareness of session objectives 1-3? 1= very much below average. 5= very much above average.

1 2 3 4 5

In your opinion, which aspects of tonight's dinner were most useful or informative?

In your opinion, which aspects of tonight's dinner were the least useful or informative?

Please, use the space below to make any additional comments or recommendations.
